# Novel outcomes in inflammatory bowel disease

**DOI:** 10.1093/ecco-jcc/jjaf040

**Published:** 2025-03-13

**Authors:** Vipul Jairath, Neeraj Narula, Ryan C Ungaro, Itzel Romo Bautista, Shashi Adsul

**Affiliations:** Department of Medicine, Division of Gastroenterology, Western University, London, ON, Canada; Alimentiv Inc., London, ON, Canada; Division of Gastroenterology, Department of Medicine and Farncombe Family Digestive Health Research Institute, McMaster University, Hamilton, ON, Canada; The Henry D. Janowitz Division of Gastroenterology, Icahn School of Medicine at Mount Sinai, New York, NY, United States; Takeda Development Center Americas, Inc., Cambridge, MA, United States; Takeda Pharmaceuticals, Inc., Cambridge, MA, United States (at the time of the analyses)

**Keywords:** inflammatory bowel disease, trial endpoints, composite endpoints

## Abstract

Inflammatory bowel diseases (IBD), Crohn’s disease (CD), and ulcerative colitis (UC) are lifelong chronic, relapsing, and remitting conditions that culminate in disease progression in many patients. Effective management of CD and UC requires consideration of both short- and long-term treatment outcomes. Historically, short-term outcomes such as clinical and endoscopic remission and symptom relief have been evaluated in clinical trials. With the expansion of treatments targeting underlying disease pathophysiology, there is the opportunity to develop management strategies that improve disease control and patients’ lives in both the short and the long term. Researchers have been examining novel outcomes for assessing the efficacy of CD and UC treatments that are important to patients, and also those that go beyond symptomatic improvements or clinical remission. These include new patient-reported outcomes for symptoms, as well as transmural/histological healing and disease clearance that can be more reflective of deeper remission states and disease modification. This review analyses published clinical studies involving patients with UC and CD treated with biologics or small molecule therapies. It highlights novel IBD endpoints employed in published clinical trials and discusses their likely value for assessing disease activity and disease modification, and as predictors of reduced risk of complications and morbidities.

## Graphical Abstract

**Graphical Abstract:**
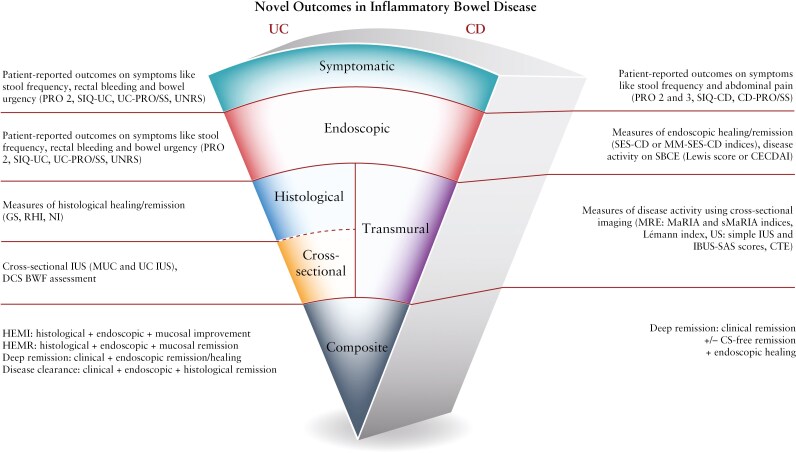

## 1. Background

Crohn’s disease (CD) and ulcerative colitis (UC) are disabling, lifelong inflammatory bowel diseases (IBD) with increasing global prevalence. The high prevalence of IBD in high-income industrialized countries of North America, Europe, and Australasia translates to a high burden of IBD in these countries.^1,2^ While the prevalence of IBD in newly industrialized countries in South America, Eastern Europe, Asia, and Africa is low, the increasing incidence of IBD reported in countries within these regions means prevalence will rise and, consequently, so will disease burden and the demand for IBD-related healthcare services.^2^

Symptoms of IBD, which include abdominal cramps and pain, diarrhea, fatigue, and weight loss,^3,4^ can have a sizable impact on patients’ health-related quality of life, negatively affecting physical and psychosocial functioning.^5^ IBD also impacts work productivity, with impairment increasing with disease activity.^6^ CD and UC are progressive conditions and their disease burden increases over time, due to the development of complications and the need for IBD-related surgery and hospitalizations. Individuals with IBD are also at increased risk of intestinal and extraintestinal cancer.^3,4,7^

Given the impact symptoms have on patients’ lives and the long-term risk of complications and morbidities, effective therapeutic management of CD and UC requires a combination of short- and long-term treatment goals, as well as objective measures to monitor disease activity. The STRIDE-II clinical practice recommendations include reducing and resolving the symptoms of active disease to provide relief to patients in the short-to-intermediate term, with healing of the mucosa and normalizing quality of life identified as targets in the longer term.^8^

Biologic and small molecule agents available to treat patients with moderate-to-severe CD and UC target the underlying pathophysiology of CD and UC and provide the potential for disease modification.^9^ Expert consensus opinions on the best endpoints for measuring disease modification and prevention of disease progression have been outlined in the SPIRIT guidelines (Table 1).^9^ However, the effects of treatments on disease modification endpoints take much longer to assess and are less suited to evaluation in prospective clinical trials. Clinical remission, encompassing symptomatic and endoscopic definitions of remission, remains the primary endpoint to evaluate the efficacy of interventions in clinical trials as per regulatory guidance from the Food and Drug Administration (FDA) and the European Medicines Agency (EMA).^10–13^

**Table 1.** Endpoints included in the SPIRIT guidelines.

PROs	Midterm complications	Long-term complications
Health-related quality of life (IBDQ-36 + SF-36)	Bowel damage in CD (Lémann index at 12-24 months)	Dysplasia or cancer (at 5 years)
Disability (IBD disability index)	IBD-related surgery (colectomy, CD-related surgery, endoscopic balloon dilation, perianal surgery at 24-36 months)	Mortality (both IBD-related and non-IBD-related mortality at 5 years)
Fecal incontinence (Jorge and Wexner [Cleveland score] at 6-12 months)	IBD-related hospitalizations (number of hospitalizations + cumulative length of stay from 12 to 24 months)	
	Disease extension in UC (macroscopic proximal disease extension from 2 to 5 years)	
	EIM from 12 to 36 months	
	Permanent stoma	
	Short-bowel syndrome	

Abbreviations: CD, Crohn’s disease; EIM, extraintestinal manifestations; IBD, inflammatory bowel disease; IBDQ, inflammatory bowel disease questionnaire; SF, short-form; UC, ulcerative colitis.

In the Randomized Evaluation of an Algorithm for Crohn’s Treatment (REACT; NCT01030809) trial, community gastroenterology practices in Belgium and Canada assigned adult patients with CD to 1 of 2 disease management approaches: early combined immunosuppression (ECI) or conventional step-care management (sequential treatment with corticosteroids, immunomodulators and anti-TNFα agents). After 12 months, the proportion of patients in corticosteroid-free remission at the practice level was similar between ECI and conventional management groups.^14^ However, the patient-level composite rate of major adverse outcomes (defined as occurrence of surgery, hospital admission, or serious disease-related complications) analyzed at 24 months was lower at ECI practices than conventional management practices, suggesting that early initiation of highly effective therapy might have a disease modification effect. In REACT-2 (NCT01698307), practices were randomized to either early combination therapy with treatment intensification to a target of absence of ulcers (>5 mm in size) or step-care with treatment intensification to a target of clinical remission.^15^ Although there was no difference between groups for the primary outcome of time to first occurrence of CD-related complications, there was a 25% reduction in the risk of CD-related complications in patients with active disease at baseline who were assigned to early combination therapy, suggesting that treating to a target of ulcer healing is more effective than symptom-based management in these patients. These 2 trials highlight the uncertainty which remains with regards to achieving disease modification using a treat-to-target strategy.

**Figure 1.**
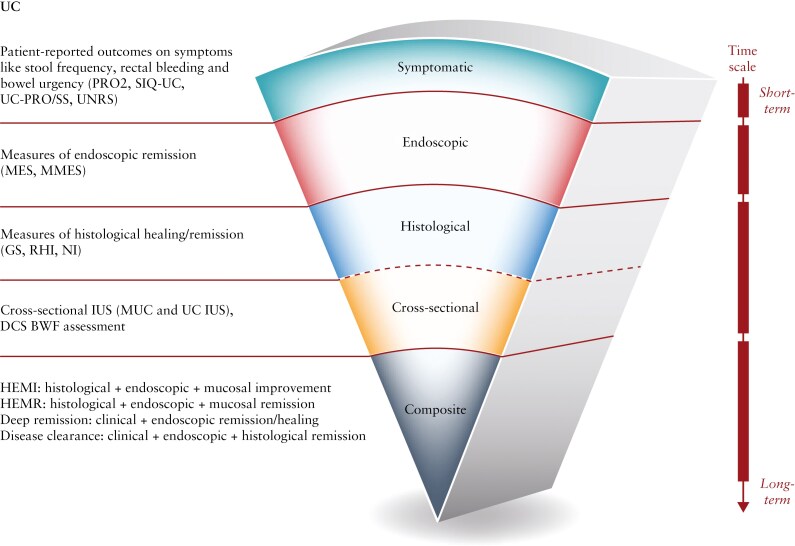
Summary of novel IBD ulcerative colitis study endpoints. Abbreviations: BWF, bowel wall flow; DCS, Doppler color signal; GS, Goebes Score; HEMI, histological, endoscopic, and mucosal improvement; HEMR, histological, endoscopic, and mucosal remission; IUS, intestinal ultrasound; PRO2, patient-reported outcome 2 (includes stool frequency and rectal bleeding components from the Mayo score); MES, Mayo Endoscopic Score; MMES, modified Mayo Endoscopic Score; MUC, Milan US Criteria; NI, Nancy Index; RHI, Robarts Histology Index; SIQ-UC, Symptoms and Impacts Questionnaire for Ulcerative Colitis; UC-PRO/SS, Ulcerative Colitis Patient-Reported Outcomes Signs and Symptoms; UC, ulcerative colitis; UC IUS, Ulcerative Colitis Intestinal Ultrasound Index; UNRS, Urgency Numerical Rating Scale.

**Figure 2.**
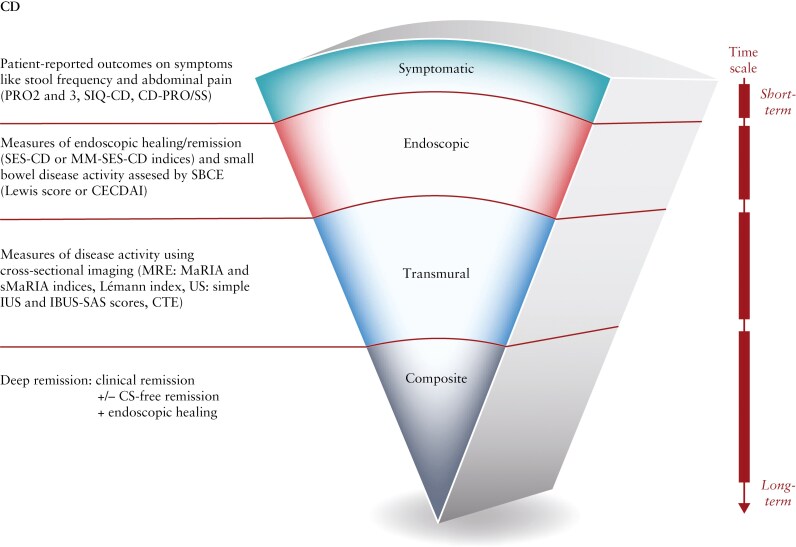
Summary of novel IBD Crohn’s disease study endpoints. Abbreviations: CD, Crohn’s disease; CD-PRO/SS, Crohn’s Disease Patient-Reported Outcomes Signs and Symptoms; CDAI, Crohn’s disease activity index, CECDAI, Capsule Endoscopy Crohn’s disease activity index; CTE, computed tomography enterography; CS, corticosteroid; IBD, inflammatory bowel disease; IBUS-SAS, International Bowel Ultrasound Segmental Activity Score; IUS, intestinal ultrasound; PRO2, patient-reported outcome 2 (includes stool frequency and abdominal pain components from the CDAI); PRO3, patient-reported outcome 3 (includes stool frequency, abdominal pain, and well-being components from the CDAI); MaRIA, Magnetic Resonance Index of Activity for CD; MM-SES-CD, modified multiplier of the SES-CD; MRI, magnetic resonance imaging; SBCE, Small Bowel Capsule Endoscopy; SES-CD, Simple Endoscopic Score for CD; SIQ-CD, Symptoms and Impacts Questionnaire for Crohn’s Disease; sMaRIA, simplified Magnetic Resonance Index of Activity for CD; US, ultrasound.

Researchers have been examining novel outcomes for assessing the efficacy of CD and UC treatments that are important to patients and ones that go beyond symptomatic improvements or clinical remission, including transmural/histological healing and disease clearance that can be reflective of deeper remission states and disease modification. The aim of this manuscript is to review clinical studies of patients with UC and CD treated with biologic or small molecule therapies that employ novel IBD endpoints (Figures 1 and 2), including patient-reported outcomes (PROs), for symptoms, histological outcomes, transmural healing, and composite endpoints.

## 2. Search strategy

A PubMed search was conducted for publications on clinical studies of patients with UC or CD receiving biologic or small molecule therapies which employed novel IBD endpoints, including those recommended in the SPIRIT guidelines (Table 1).

## 3. Rationale and practicalities of novel outcomes in UC

### 3.1. Novel PROs-symptomatic PROs as endpoints in UC studies

Recently there has been a drive to develop simplified, more patient-centric outcomes. An international consensus initiative set up to develop a set of core outcomes for randomized controlled trials in IBD (CORE-IBD) recommended that the adapted 9-point Mayo Clinic Score combining PROs of stool frequency (SF) and rectal bleeding (RB) (reflecting the hallmark of UC symptoms collectively known as PRO2) and modified Mayo Endoscopic Subscore (MES) should be used for randomized controlled UC trials. An RB score of 0 and an SF of ≤1 are considered clinical remission.^16^ Developing a robust PRO that also conforms to the FDA-endorsed pathway of PRO development is a long and rigorous process involving multiple development and validation steps. PRO2 was validated in 2015 as an interim PRO to allow for the continued evaluation of therapies while the FDA was evaluating endpoints for clinical trials intended to support approval of new IBD treatments.^17^ Two UC-specific PRO instruments, the Symptoms and Impacts Questionnaire for Ulcerative Colitis (SIQ-UC) and the Ulcerative Colitis Patient-Reported Outcomes Signs and Symptoms (UC-PRO/SS), are currently in development following FDA and International Society for Pharmacoeconomics and Outcomes Research (ISPOR) best practice recommendations.^18,19^

CORE-IBD also recommended that bowel urgency should be captured as a core outcome since patients consider it to be the most debilitating symptom.^16^ Bowel urgency has a substantial negative impact on quality of life and patient psychosocial functioning as the most common and disruptive UC symptom.^20^ As well as being independently associated with reduced quality of life, urgency is also associated with future risk of hospitalizations, steroid prescription, and colectomy.^21^ An online survey of patients and healthcare professionals showed that bowel urgency was 1 of the top 3 concerns for patients, along with diarrhea and increased SF.^22^ Healthcare professionals did not include bowel urgency in the top 3 priorities, highlighting a communication gap between patients and healthcare professionals. A single-item PRO measure to assess the severity of bowel urgency in adults with UC, the Urgency Numerical Rating Scale (UNRS), has recently been developed in accordance with FDA PRO best practice guidance.^23^

### 3.2. Histological remission as an endpoint in UC studies

Endoscopic remission is associated with longer-term improvements in UC clinical outcomes and is recommended as an endpoint for clinical trials.^11,16^ Efforts to improve the prognostic capability of endoscopic scoring and provide more information on the extent of mucosal inflammation have led to the development of the modified MES (MMES), which was recently shown to be predictive for long-term clinical outcomes when tested in a prospective observational study.^24^ Histological assessment has been proposed as a more accurate and deeper measure of disease activity than endoscopic remission.^25^ Histological activity and histological remission have been associated with negative and positive long-term clinical outcomes, respectively. Among patients with endoscopic remission, histological activity has been independently associated with clinical relapse in observational research.^26,27^ Over the long term, the risk of clinical relapse, surgery, and risk of hospitalization were significantly higher in patients with active histological disease than in patients in histological remission.^28^ Histological remission was associated with a decreased risk of disease relapse/exacerbation, decreased risk of colectomy, and decreased corticosteroid (CS) use when compared with patients with histological activity.^29^ This endpoint has also been demonstrated to be associated with reduced rates of hospitalization.^30^

CORE-IBD recommendations include incorporation of histological remission as a core outcome in UC clinical trials.^16^ Histological remission, also called healing in UC, is also recognized by the STRIDE-II consensus panel as an adjunctive treatment target.^8^ Although recognized as a goal for treatment, the rates of achieving histological remission range from 15.0% to 44.9%, and vary according to therapeutic drug class and patient population. The highest reported rates for histological remission have been observed with topical aminosalicylates, although comprehensive data on rates achieved with biologics and small molecules are currently lacking.^31^

The most widely used and well-validated histological assessments are the Geboes score (GS), the Robarts Histopathology Index (RHI), and the Nancy Index (NI).^32,33^ For all 3 indices, intra- and interobserver agreement is reported as excellent, and they are reliable and responsive tools to measure disease activity and evaluate treatment response.^32–34^ Before histological healing can be considered a widely accepted treatment endpoint, consensus agreement on validated definitions for histological healing and remission is required, but specifically achievement of this surrogate endpoint will need to be associated with superior meaningful patient outcomes. Evidence-based protocols regarding the timing, location, and number of biopsies are also needed.^32,33,35,36^ As will be discussed, histological remission is more often assessed as a component of a composite endpoint.

### 3.3. Cross-sectional measures using intestinal ultrasound as an endpoint in UC studies

There is growing evidence that intestinal ultrasound (US) could be used to determine disease activity and to monitor therapeutic response in patients with active UC. Using colonoscopy as the reference standard, bowel wall thickening (BWT) > 3 mm shown on intestinal US was shown to be an independent predictor of endoscopic activity; a cutoff of 2.1 mm was used to discriminate between inactive and active endoscopic disease activity with >80% sensitivity and specificity. Bowel wall flow (BWF) on Doppler color signal (DCS), a measure of vascularization and disease activity, was also shown to be an independent predictor of endoscopic disease activity and identification of any DCS was associated with the presence of endoscopic disease activity.^37,38^

The Milan US Criteria (MUC) and the UC Intestinal Ultrasound (UC IUS) Index have been developed as scores of intestinal US activity, with the MUC being the more developed of the 2. The MUC uses a score (1.4 × BWT plus 2 × BWF) to assess and grade UC disease activity; the accuracy of these scoring parameters for detecting active vs nonactive disease has been validated.^37,39^ The point-based UC IUS index, which grades 4 parameters (BWT, DCS, abnormal haustration, and fat wrapping) on a 7-point scale, remains to be validated.^38,40^ Although not a formal therapeutic target recommended by the STRIDE-II consensus,^8^ IUS is a valuable technique to assess intestinal inflammation in patients with UC, and is currently being used as an intermediate target in clinical care. Further evidence is needed to validate its use in this setting and as a primary or longer-term outcome for clinical trials. Notable limitations include that mucosal rather than transmural involvement makes it more difficult to detect bowel lesions with US. Also, the location of the rectum deep within the pelvis makes this area difficult to assess using transabdominal US.^41^

### 3.4. Novel composite outcomes in UC studies

Novel composite endpoints in UC include histological-endoscopic mucosal improvement (HEMI) and the more stringent histological-endoscopic mucosal remission (HEMR), deep remission, and the more stringent endpoint of disease clearance. HEMI has been defined as an MES ≤1 and a GS of ≤3.1. HEMR or deep mucosal healing has been defined as MES = 0 or MES = 1 excluding friability and GS <2.^42^ Deep remission in UC is considered to be concurrent clinical remission and endoscopic remission or mucosal healing, while disease clearance is a more stringent composite outcome of clinical, endoscopic, and histological remission.^43^ While there is no definitive definition for deep remission, members of the International Organization for the Study of IBD proposed a quantifiable definition for disease clearance^44,45^: “a composite outcome including simultaneous clinical remission (partial Mayo score of 0), endoscopic remission (MES of 0) and histologic remission (NI of 0).” Adoption of this definition would enable standardized evaluation of disease clearance as an outcome.^43,45^

Reaching this composite endpoint, where no disease activity is observed at the time point of assessment, has recently been proposed as the ultimate goal in the treatment of UC in terms of lowering the risk of UC‐related complications and reducing indirect costs.^43,46^ Definitive evidence on whether disease clearance can prevent or delay UC-related complications, to a greater extent than the targets of deep remission or symptomatic remission alone, is currently being evaluated in the VERDICT trial. This prospective, randomized trial employs treatment algorithms featuring the early use of vedolizumab to attain 1 of 3 treatment targets: CS-free symptomatic remission, CS-free symptomatic remission plus endoscopic remission, or CS-free symptomatic remission plus endoscopic remission plus histologic remission, compared using the endpoint of time to reach a UC-related complication.^47^

### 3.5. Novel steroid-sparing endpoints in UC studies

The CORE-IBD consensus was that CS-free remission was important to include as a maintenance outcome in UC studies; the sensitivity of UC symptoms to CS treatment also suggested a need to stipulate clear CS dosing rules in the induction and maintenance treatment phases. No consensus was reached for the definition of CS-free remission.^16^ The primary objective of the ongoing VERDICT study is to compare the target of CS-free symptomatic and endoscopic and histologic remission with that of CS-symptomatic remission alone, in terms of time to UC-related complications, where “CS-free” is defined as not receiving oral CS at the time of treatment target assessment at weeks 16, 32, and 48.^47^ The ELEVATE UC 52 trial included 3 maintenance phase outcomes requiring no CS exposure in the 12 weeks prior to week 52 (CS tapering began after the 12-week induction phase). In addition to CS-free clinical remission, the endpoint of CS-free endoscopic improvement included patients achieving an MES of ≤1 (excluding friability) without CS for the last 12 weeks of maintenance treatment. The CS-free symptomatic remission endpoint included patients achieving an SF subscore of 0 (or =1 with a ≥1-point decrease from baseline) and RB subscore of 0.^48^

## 4. Rationale and practicalities of novel outcomes in CD

### 4.1. Novel symptomatic PROs as endpoints in CD studies

The CD Activity Index (CDAI) has been used as the primary outcome measure for evaluation and approval of treatments in CD trials for over 40 years. The PRO diary components of the CDAI, which capture liquid stools and abdominal cramps, the hallmark of CD symptoms, have been validated as an interim PRO, termed PRO2. Incorporating SF and abdominal pain (AP) symptoms, PRO2 was also recommended by CORE-IBD.^16^ A 3-item PRO (PRO3) incorporating general well-being in addition to SF and AP CDAI score components has also been validated.^49^ Although PROs specific for use in CD are not yet available, some are currently in development, including the Symptom and Impacts Questionnaire for Crohn’s Disease and the CD-PRO/SS diary.^18,50^

### 4.2. Histological outcomes as endpoints in CD studies

In contrast to UC, histological healing in CD is not considered a core outcome measure by the CORE-IBD Initiative nor a treatment target according to the STRIDE-II recommendations.^8,16^ Histological assessment is less suitable as an outcome measure in CD because of the segmental, transmural nature of condition, with “patchy” distribution of inflammation that can occur disparately in any part of the gut. These features are likely to reduce the reliability of histopathology for assessment of disease activity in CD.^51^ Moreover, there is a lack of well-validated and reliable scoring tools for CD histological assessment.^8,52^ The global histologic disease activity score (GHAS) is a widely used system that allows the ileal and colonic regions to be graded separately. UC indices like the GS and RHI are also used to grade histopathology in CD. Of note, it is presently unclear which histologic features are the most relevant to measure in terms of disease pathophysiology or treatment outcomes in CD, although UC scoring indices have acceptable reliability in this setting.^53^ The evidence linking histologic activity in CD to disease outcomes is currently insufficient to justify intensified immunosuppressant medications to reach histological remission as a treatment target.^8,44,51^ There is also the question of whether histological remission is achievable with current treatments. A 2023 meta-analysis of randomized controlled trials in CD reported 38% of patients achieving histological remission during induction treatment assessed at 4 or 12 weeks.^54^ Nevertheless, as in UC, there may turn out to be added value to achieving histological remission.^51^

### 4.3. Endoscopic outcomes as endpoints in CD studies

The STRIDE-II consensus did recommend endoscopic healing as a treatment goal in CD.^8^ Endoscopic healing does not necessarily indicate the absence of histologic inflammation, as up to one-third of biopsies from patients with CD with endoscopically healed mucosa may show evidence of histologic disease.^55^ Nevertheless, patients who achieve endoscopic remission have improved long-term disease outcomes.^56^ Recently, because of the differences in Simple Endoscopic Score for CD (SES-CD) definitions used for endoscopic remission, there has been a move towards targeting endoscopic scoring thresholds, which have been associated with lower risk of long-term disease progression.^57^ The modified multiplier of the SES-CD (MM-SES-CD) was designed by weighting individual SES-CD parameters according to their ability to predict endoscopic remission, as determined by logistic regression modeling.^58^ A post hoc analysis of 61 patients in the CALM long-term extension study demonstrated that patients with endoscopic remission (defined as SES-CD <4 or MM-SES-CD <22.5) were less likely to have disease progression over the long term than those not achieving these targets.^59^

Assessment of the small bowel in patients with CD is necessary because complete visualization of the entire length of the small bowel may have a significant impact on prognosis and potential therapeutic implications.^60^ Small bowel capsule endoscopy (SBCE) is particularly useful in areas of the GI tract that are not accessible to conventional endoscopy.^60^ The STRIDE-II consensus recommends use of capsule endoscopy when sigmoidoscopy or colonoscopy is not feasible.^8^ In addition, the European Society of Gastrointestinal Endoscopy (ESGE)^61^ and European Crohn’s and Colitis Organisation (ECCO)^62^ guidelines recommend the use of validated endoscopic scoring indices such as the Lewis score^63,64^ or the capsule endoscopy CDAI (CECDAI) score^65–67^ for the classification of inflammatory activity in patients with CD undergoing SBCE.^68^ These validated scores standardize descriptions of lesions and help quantify disease activity and severity.^68^ SBCE can be seen as complementary to magnetic resonance enterography (MRE) because MRE assesses transmural involvement while SBCE allows direct visualization of the mucosal surface of the small bowel to detect mucosal lesions.^60^ In a treat-to-target strategy, SBCE could be useful for refining disease location and prognosis, assessing mucosal healing in patients receiving treatment, and monitoring patients in the post-operative setting.^60^ Capsule endoscopy data, reported recently from the CURE-CD randomized controlled trial, showed that a treat-to target strategy in high-risk patients with active inflammation was associated with lower rates of clinical relapse compared with standard of care.^69^ Although many randomized controlled trials have not included assessment of the small bowel in their design, this should be considered for future trials given the prognostic value of small bowel lesions. In addition, the usefulness and reliability of SBCE need to be confirmed in randomized controlled studies.

### 4.4. Transmural healing as an endpoint in CD studies

Transmural healing in CD refers to healing of all layers of the bowel and is considered an aspirational treatment target by the STRIDE-II consensus panel.^8^ Transmural healing is associated with substantial improvements in long-term CD-related outcomes for patients with CD. A systematic literature review identified 17 studies on transmural healing in CD. This outcome was significantly associated with clinical remission in 3 out of 6 studies and with decreased hospitalization rate in 4 out of 7 studies. Patients with transmural healing also had a significantly decreased risk of CD-related surgery compared with patients without transmural healing in 9 out of 10 studies.^70^ While there are demonstrable benefits of transmural healing, this outcome can be difficult to achieve, with reported rates ranging from 14% to 42% following treatment.^70^ There is no single validated definition for transmural healing to date, and proposed definitions involve normalization of the BWT of inflamed bowel sections.^71^

### 4.5. Deep remission as an endpoint in CD studies

The initial concept of deep remission in CD was considered to be the resolution of clinical symptoms and mucosal healing.^44,72^ Deep remission (defined as the absence of mucosal ulceration and a CDAI score < 150) achieved at week 12 by patients receiving adalimumab treatment in the EXTEND trial was associated with significantly fewer adalimumab treatment adjustments, hospitalizations, and CD-related surgeries assessed at week 52, compared with patients who did not achieve deep remission by week 12.^73^ In the CALM study, deep remission (defined as a CDAI score of <150, a CD Endoscopic Index of Severity score of <4 with no deep ulcerations, and no CS for ≥8 weeks) was associated with an 81% decrease in risk of disease progression over a median of 3 years.^74^

### 4.6. Novel steroid-sparing endpoints in CD studies

CORE-IBD reached a consensus to include CS-free remission as part of a co-primary endpoint in CD maintenance studies. There was also interest in earlier CS tapering during induction treatment to mitigate the risk of CS-related adverse events, and because endoscopic assessments to detect early mucosal improvements are increasingly being included in co-primary endpoints for CD induction studies.^16^ In a post hoc analysis of the SEQUENCE study, CS-free outcomes of clinical remission or endoscopic remission were examined at week 48 among patients receiving CS at baseline (after a mandatory CS taper at week 2). The analysis included assessment of patients receiving risankizumab or ustekinumab who achieved CS-free outcomes for 90 days prior to week 48.^75^

## 5. Clinical studies employing novel endpoints in UC

Data from novel, objectively measured outcomes used in clinical studies of patients with UC are shown in Table 2.

**Table 2.** Studies of biologic and small molecule therapies using novel IBD objectively measured outcomes in UC.

Study	Treatment	Key study details and findings
Histological activity, including histological remission alone and composite endpoints of HEMI and HEMR		
VARSITY^76^ Histological remission was a prespecified endpoint in an RCT in the Phase 3 VARSITY study	Vedolizumab vs adalimumab	Histological remission = GS < 2 or RHI score ≤ 2 at week 14 and week 52 (prespecified exploratory endpoints)Statistically significant higher remission rates were observed with vedolizumab vs adalimumab at week 14 and week 52 on the GS and RHI.Histological remission (GS < 2) vedolizumab vs adalimumab Week 14: 16.7% vs 7.3%; difference 9.4% (95% CI, 4.9%-13.9%); *P* < .0001 Week 52: 29.2% vs 8.3%; difference 20.9% (95% CI, 15.6%-26.2%); *P* < .0001 Histological remission (RHI score ≤ 2) vedolizumab vs adalimumab Week 14: 25.6% vs 16.1%; 9.5% (95% CI, 3.8%-15.2%); *P* = .0011 Week 52: 37.6% vs 19.9%; difference 17.6% (95% CI, 11.3%-23.8%); *P* < .0001
LUCENT I and II^77^Histological and histological-endoscopic endpoints were included at outcomes in Phase 3 induction and maintenance trials	Mirikizumab	Histological remission = GS < 2Endoscopic remission = MES of 0 or 1 (excluding friability)HEMI = histological improvement + endoscopic remissionHEMR = histological remission + endoscopic remissionAt week 12, mirikizumab vs placebo Histological remission: 29% vs 16%; common risk difference 13.7%; *P* < .001 HEMI: 27% vs 14%; common risk difference 13.4%; *P* < .00001 HEMR: 22% vs 11%; common risk difference 11.3%; *P* < .001 At week 40, mirikizumab vs placebo Histological remission: 49% vs 25%; common risk difference 22.5%; *P* < .001 HEMI: 48% vs 22%; common risk difference 23.9%; *P* < .001 HEMR: 43% vs 22%; common risk difference 19.9%; *P* < .001 Histological remission, HEMI, and HEMR at week 12 were associated with corticosteroid-free remission, clinical remission, and symptomatic remission at week 40
ELEVATE UC 52 and ELEVATE UC 12^48^Histological-endoscopic were included as outcomes endpoints for Phase 3 trials	Etrasimod	HEMR = Histological remission (GS < 2) + endoscopic remission MES ≤ 1, without friabilityIn ELEVATE UC 52 Week 12: 21% (58/274) patients treated with etrasimod vs 4% (6/135) patients treated with placebo achieved HEMR; difference 16.9% (95% CI, 10.8-23.0); *P* < .0001 Week 52: Difference between etrasimod vs placebo on HEMR of 18.4% (95% CI, 11.4-25.4); *P* < .0001 ELEVATE UC 12 at week 12 16% (36/222) patients treated with etrasimod vs 9% (10/112) patients treated with placebo achieved HEMR; difference 7.4% (95% CI, 0.5-144); *P* = .036
GEMINI I and LTS^78^Study carried out at University Hospitals Leuven using biopsy samples from patients enrolled in the Phase 3 GEMINI I study and its open-label long-term extension study at that center	Vedolizumab	Histological mucosal healing = GS 0 or 1 and Endoscopic mucosal healing = MES of 0 or 122 patients treated with vedolizumab achieved mucosal healing, and of these 12 (55%) also showed histological healing, that is, HEMR at the timepoints studied (3/6 at week 6, 2/3 at week 12, and 7/12 at week 52)
UNIFI Phase 3 clinical trials pooled analysis to examine clinical relevance of histological improvements alone and with endoscopic improvement^79^	Ustekinumab	Histologic improvement = GS ≤ 3.1Endoscopic improvement = MES of 0 or 1.HEMI = histologic + endoscopic improvementClinical remission = Mayo score ≤ 2 points, with no individual subscore > 1 At week 8, following ustekinumab induction Patients with histologic improvement (283/816) ~20 times more likely to achieve week 8 clinical remission vs those without histologic improvement (OR 19.9; 95% CI, 10.7-39.5) Patients with histologic improvement at week 8 were ~12 times more likely to have endoscopic improvement vs patients without histologic improvement (OR 11.9 [95% CI, 8.0-17.9]) At week 44 Clinical remission at week 44 was achieved in 54% (76/140) patients with histological improvement at week 8 after ustekinumab induction vs 40% (49/124) in patients without histological improvement at week 8 (*P* = .0191) Considering patients with positive outcomes at week 44, 61% (56/92) patients with HEMI after induction achieved clinical remission, vs 34% (24/71) of patients with histologic improvement alone after induction (*P* = .0009)
U-ACHIEVE induction and U-ACCOMPLISH indication and U-ACHIEVE^42^ pooled analysis to examine to assess the clinical relevance of achieving HEMI and HEMR	Upadacitinib	HEMI = GS ≤ 3.1 and MES of 0 or 1HEMR = GS < 2 and MES of 0CS-free remission = 90-day CS-free clinical remission (total Mayo score ≤ 2 no subscore > 1)The proportion of patients who achieved CS-free remission at week 52 among patients with no HEMI (*n* = 197) was 6%, with HEMI without HEMR (*n* = 78) was 80%, and with HEMR (*n* = 45) was 89% (*P* < .001 comparison vs no HEMI for both HEMI without HEMR and HEMR)
Intestinal ultrasound		
Prospective observational study^80^	Standard of care	BWT assessed by intestinal US and vascularization within the affected bowel wall areas was assessed by DCSThe percentage of patients with increased BWT was reduced significantly from 89.3% of 224 patients at baseline to 32% at week 12 (*n* = 178) in the sigmoid colon and from 83.0% at baseline to 37.6% at week 12 in the descending colonImprovements in vascularization observed were maintained up to week 12 in both the sigmoid and descending colon
Longitudinal prospective study^81^	Tofacitanib	BWT measured by intestinal USBWT was shown to be significantly lower in patients with endoscopic improvement compared with patients without endoscopic improvement after 8 weeks of tofacitinib treatment (analysis of 27 patients)
Prospective pilot study^82^	Vedolizumab	Vascularization of the bowel wall was assessed with high-frequency ultrasound using DCS in 18 patients at baseline and 14 weeks after vedolizumab treatmentNine of 18 patients (11 with CD, 7 UC) responded to vedolizumab treatment and had a significant decrease in bowel wall vascularization
Composite endpoint of deep remission		
Post hoc analysis GEMINI 1^83^	Vedolizumab	Four deep remission endpoints were defined from high to low stringency:1. Endoscopic remission + symptom improvement: MES = 0; RB = 0; decrease or no change in baseline SF score4. Endoscopic + symptomatic improvement: MES ≤ 1; RB = 0; SF = ≤1Vedolizumab Q8W treatment group (*n* = 122 patients) or vedolizumab Q4W group (*n* = 125) had significantly higher deep remission rates than the placebo group (*n* = 126) at week 52, regardless of deep remission definition. Most stringent definition of deep remission—(1) endoscopic remission and symptomatic improvement: vedolizumab Q8W 27.0% or Q4W 28.0% vs placebo 8.7% (*P* < .0001 for both comparisons) Least stringent definition of deep remission—(4) endoscopic and symptomatic improvement: Q8W 43.4% or Q4W 43.2% vs placebo 15.9% (*P* < .0001 for both)
Multicenter, observational, prospective study^84^	Adalimumab	Deep remission (evaluated as a secondary endpoint) = clinical remission (pMS ≤ 2 plus blood-in-the-stool assessment at value 0) + mucosal healing (MES of 0 or 1)Deep remission was achieved in 43.4% (23/53) and 58.5% (31/53) of patients at week 8 and week 52 of adalimumab treatment, respectively
Retrospective review of VICTORY Consortium data^85^	Vedolizumab	Deep remission = clinical remission (complete resolution of all UC-related symptoms) + endoscopic remission (MES of 0)Among 321 patients (71% anti-TNFα experienced, median follow-up 10 months), overall cumulative rates of deep remission were 14% at 6 months and 30% at 12 months of vedolizumab treatment
Multicenter, retrospective, observational cohort study using propensity score weighted comparisons^86^	Vedolizumab vs TNFα-antagonists	Deep remission = clinical remission (resolution of diarrhea, RB and urgency) + endoscopic remission (MES of 0 or 1)CS-free deep remission = no CS within 1 month of clinical remission + endoscopic remissionAnalyzed 454 vedolizumab-treated and 268 anti-TNFα-treated patients with UC. Vedolizumab-treated patients were more likely to achieve deep remission (HR 1.7 [95% CI, 1.0-2.8]; *P* = .06) and CS-free deep remission (HR 2.8 [95% CI, 1.5-5.3]) than anti-TNFα-treated patients
Composite endpoint of disease clearance		
VARSITY, post hoc analysis of Phase 3 trial^87^	Vedolizumab vs adalimumab	Disease clearance = a composite outcome based on clinical remission (pMS ≤ 2 and no individual subscore > 1 excluding sigmoidoscopy subscore) + endoscopic improvement (MES of ≤1) + absence of active histologic disease (RHI < 5)More patients treated with vedolizumab than adalimumab achieved disease clearance at week 52 (vedolizumab: 112/383, 29.2% [95% CI, 24.7-34.1] vs adalimumab: 63/386, 16.3% [95% CI, 12.8-20.4])
Multicenter retrospective real-world cohort.^88^	Multiple treatments, most commonly Thiopurines, infliximab, Vedolizumab, and adalimumab	Disease clearance = clinical remission (pMS ≤ 2, with no subscore > 1) + endoscopic remission (MES = 0) + histological remission (NI = 0)22.1% (109/494) of patients had disease clearance after inductionPatients with disease clearance had a significantly lower risk of negative outcomes vs those without disease clearance; negative outcomes were any escalation of medical therapy, UC‐related hospitalization, UC‐related surgery, colorectal dysplasia/neoplasia, and death (HR 0.22 [95% CI, 0.10-0.48]; *P* < .001), hospitalization (HR 0.20 [95% CI, 0.09-0.45]; *P* < .001), and surgery (HR 0.14 [95% CI, 0.03-0.59]; *P* = .007)

Abbreviations: BWT, bowel wall thickness; CD, Crohn’s disease; CI, confidence interval; DCS, Doppler color signal; GS, Goebes Score; HEMI, histologic endoscopic mucosal improvement; HEMR, histologic endoscopic mucosal remission; HR, hazard ratio; IBD, inflammatory bowel disease; MES, Mayo Endoscopic Score; NI, Nancy index; OR, odds ratio; pMS, partial Mayo score; Q4W, every 4 weeks; Q8W, every 8 weeks; RCT, randomized controlled trial; RHI, Robarts Histology Index; TNFα, tumor necrosis factor alpha, UC, ulcerative colitis; US, ultrasound.

### 5.1 PROs as endpoints

Post hoc analyses of data from pivotal trials using the PRO2 endpoint (defining symptomatic clinical remission as an RB score of 0 and SF of ≤1) have been conducted for vedolizumab and ustekinumab biologics and the small molecules filgotinib and tofacitinib (Table 3). Speed of response has been a focus for 3 of these analyses, with around 20% of patients receiving active treatment achieving PRO2 clinical remission before or at 2 weeks posttreatment.^89–91^ Maintenance of symptomatic remission was the focus on the analysis with tofacitinib, which showed 98.3% of patients in PRO2 clinical remission at the end of the maintenance phase, with nearly half of them (48.0%) retaining that status 48 weeks later following open-label treatment.^92^ A prospective cohort study of anti-TNFα experienced patients initiated either on vedolizumab (*n* = 72) or tofacitinib (*n* = 33) treatment included PRO2 as a secondary endpoint. The study reported no difference in the proportion of patients achieving PRO2 for vedolizumab vs tofacitinib by month 6 of treatment.^93^

**Table 3.** Studies of biologic and small molecule therapies using novel patient-reported outcomes in UC.

Study	Treatment	Key study details and findings
Rectal bleeding plus stool frequency (PRO2)		
Post hoc analysis of GEMINI 1, GEMINI 2, and GEMINI 3^89^	Vedolizumab	PRO2 clinical remission = RB of 0 and SF ≤ 1A significantly higher proportion of patients achieved PRO2 clinical remission in the vedolizumab group (*n* = 225) than the placebo group (*n* = 149) at weeks 2, 4, and 6 overall and in anti-TNFα-naive patient subgroups vedolizumab-treated (*n* = 130) and placebo-treated (*n* = 76). No treatment differences among anti-TNFα-experienced patients.Vedolizumab vs placebo, overall Week 2: 19.1% vs 10.1%, difference adjusted change 9.0% (95% CI: 2.0-16.1) Week 4: 28.0% vs 14.8%, difference adjusted change 13.2% (95% CI: 5.1-21.4) Week 6: 33.8% vs 16.8% difference adjusted change 17.0% (95% CI: 8.4-25.6)
Post hoc analysis of UNIFI^90^	Ustekinumab	PRO2 clinical remission = RB of 0 and SF ≤ 1At week 2, ustekinumab-treated patients in the 130 mg IV (*n* = 320) and ~6 mg/kg IV (*n* = 322) dose groups achieved a significantly higher rate of PRO2 clinical remission (20.0%; *P* = .015) and (20.2%; *P* = .012) compared with (12.9%) for patients in the placebo group (*n* = 319)The percentage of patients achieving PRO2 clinical remission increased from baseline through week 16 for both ustekinumab groups
Post hoc analysis SELECTION trial^91^	Filgotinib	PRO2 clinical remission = RB of 0 and SF ≤ 1PRO2 clinical remission was significantly higher in patients treated filgotinib 200 mg (*n* = 262) vs placebo (*n* = 142) by day 9 in biologic-naive patients (18.8% vs 9.5%; *P* = .0144) and by day 7 in biologic-experienced patients (10.7% vs 4.2%; *P* = .0155)
Post hoc analysis of OCTAVE clinical program^92^	Tofacitanib	PRO2 clinical remission = RB of 0 and SF ≤ 1After 52-week maintenance tofacitinib 5 mg twice daily treatment in the OCTAVE Sustain study, 172 patients in remission continued treatment in the 4-year OCTAVE Open study; 84/172 (48.0%) were in PRO2 clinical remission by month 48
Prospective cohort study^93^	Vedolizumab and tofacitinib	PRO2 clinical remission = RB of 0 and SF ≤ 1 as a secondary endpointNo difference between vedolizumab-treated (*n* = 72) and tofacitinib-treated (*n* = 33) patients achieving PRO2 remission at month 6
Bowel urgency		
Post hoc analysis of induction study U-ACHIEVE^94^	Upadacitinib	Bowel urgency was recorded daily by the patient via electronic diary reporting on the previous 24 hoursAt week 8, a higher proportion of patients who received upadacitinib 15-45 mg vs placebo-treated patients reported no bowel urgency Greatest improvement for patients receiving upadacitinib 45 mg QD (*n* = 56); 46.4% reporting no bowel urgency at week 8 compared with 8.7% for placebo (*n* = 46), a 37.7%; difference (95% CI, 18.1-54.0); *P* ≤ 0.001
Post hoc analysis of U-ACHIEVE and U-ACCOMPLISH^95^	Upadacitinib	Bowel urgency was recorded daily by the patient via electronic diary reporting on the previous 24 hoursA larger proportion of upadacitinib-treated patients (*n* = 660) reported no bowel urgency compared with the placebo group (*n* = 328) at weeks 2-8 and at week 52Percent of patients reporting no bowel urgency induction treatment, upadacitinib vs placebo Baseline: ~8% both groups Week 2: 35.8% vs 13.7%; *P* < .001 Week 4: 44.3% vs 16.5%; *P* < .001 Week 6: 46.9% vs 19.0%; *P* < .001 Week 8: 51.1% vs 23.8%; *P* < .001 Maintenance treatment upadacitinib 15 mg and 30 mg vs placebo Week 8: 64.9% and 64.3% vs 49.7%; *P* < .01 Week 52: 56.1% and 63.6% vs 17.4%; *P* < .001
LUCENT I and LUCENT II^96^	Mirikizumab	Bowel urgency measured on the UNRS from daily patient-recorded information on the severity of bowel urgencyAt week 12, reduction from baseline UNRS (least squares mean ± SE) was −2.59 ± 0.08 in mirikizumab-treated patients (*n* = 868) vs −1.63 ± 0.1 for the placebo group (*n* = 294); *P* < .001At week 52, mirikizumab induction responders re-randomized to mirikizumab (*n* = 365) or placebo (*n* = 179) reported a significantly greater mean UNRS change from induction baseline in mirikizumab-treated patients −3.80 ± 0.14 than the placebo group −2.74 ± 0.20; *P* < .001
Subset of patients with proctitis in ELEVATE UC 52 and ELEVATE UC 12^97^	Etrasimod	Bowel urgency measured on the UNRS in patients with isolated proctitisAt week 12, reduction from baseline UNRS (least squares mean) was −2.76 in etrasimod-treated patients (*n* = 35) vs −0.16 in the placebo group (*n* = 16), difference −2.60 (95% CI: −4.47-0.73); *P* < .007. No treatment-related differences at week 52

Abbreviations: IV, intravenous; QD, once daily; RB, rectal bleeding; SF, stool frequency; UNRS, Urgency Numerical Rating Scale.

The effect of treatment on bowel urgency symptoms has been examined in 2 separate post hoc analyses of Phase 3 upadacitinib clinical studies using data collected from patient electronic diaries (Table 3). Upadacitinib was more effective than placebo at alleviating bowel urgency symptoms.^94,95^ The new Urgency Numerical Rating Sale has been used as an endpoint in the mirikizumab clinical trials LUCENT-1 and LUCENT-II and the etrasimod clinical trials ELEVATE UC 52 and ELEVATE UC 12 (Table 3). Statistically significant improvements compared with placebo on this scale have been observed for both active treatments.^96,97^

### 5.2 Histological remission and composite histological-endoscopic endpoints of HEMI and HEMR

Pivotal clinical trials for 3 drugs—vedolizumab, mirikizumab, and etrasimod—have reported data on histological and/or the novel composite histological-endoscopic outcomes, HEMI and HEMR. In VARSITY,^76^ higher histological remission rates were achieved for vedolizumab against the active comparator adalimumab at week 14 and week 52, when measured using both the GS and RHI. Statistically significant higher rates of remission vs placebo were noted for mirikizumab at weeks 12 and 40 using the GS in the LUCENT clinical trials program.^77^ As well as histological remission, HEMI and HEMR were included as outcomes in LUCENT I and II, and similar to histological remission, treatment differences were statistically significant in favor of mirikizumab for both these endpoints. HEMR was included as an outcome in the 12-week induction trial of etrasimod (ELEVATE UC 12) and the 12-week induction plus 40-week maintenance trial of etrasimod (ELEVATE UC 52). There were significantly higher proportions of patients achieving HEMR in the etrasimod treatment groups vs placebo at weeks 12 and 52.^48^ One of the study centers that took part in the vedolizumab GEMINI Phase 3 study examined biopsy samples from their patients. The investigators found that, of the 22 patients with endoscopic remission, 12 (55%) also showed histological remission (ie, HEMR).^78^ The clinical relevance of reducing histological inflammation with biologics and small molecules has also been examined using clinical trial data. Histological improvement, HEMI, and HEMR have been associated with CS-free remission, clinical remission, and symptomatic remission.^42,77,98^ An interim analysis of data from the VERDICT study, evaluating optimal treatment targets in patients with moderate-to-severe UC, recently reported on 212 patients with observed data assigned to the treatment target of CS-free disease clearance; defined as symptomatic remission (Mayo RB subscore = 0) + endoscopic improvement (MES ≤ 1) + histologic remission (GS < 2B.0). Patients received IV vedolizumab 300 mg following a treatment algorithm featuring early vedolizumab treatment. At week 16, 86 (41%) of patients achieved their assigned target of CS-free disease clearance, including 77/186 (41%) biologic-naive and 9/26 (35%) biologic-experienced patients.^99^

### 5.3. Cross-sectional imaging to measure disease activity in CD

Cross-sectional imaging techniques include computed tomography enterography (CTE), MRE, and intestinal US.^40^ Although CTE is widely and routinely available, use of ionizing radiation makes it unsuitable for the serial examinations required for IBD management, so its principal use is in diagnosis.^40^ MRE is performed after administration of oral contrast medium to better visualize intestinal loops. MRE activity scores have been developed as a means of obtaining objective and standardized reports of findings. The Magnetic Resonance Index of Activity for CD (MaRIA) was the first and best validated of these; however, practical limitations, including the time needed to calculate MaRIA scores, has resulted in the development and validation of a simplified version, sMaRIA, which can measure CD disease activity, severity, and response to therapy.^40,100,101^ Both MaRIA and sMARIA scores have demonstrated good interobserver agreement and responsiveness to change.^102,103^ The Lémann index, developed as a tool for assessing cumulative structural bowel damage in CD and damage progression over time, was recently updated and externally validated in a prospective, multicenter, cross-sectional observational study. The results demonstrated good correlation with expert investigator assessment and the index was considered useful for assessing midterm CD complications in disease modification trials, especially with centralized reading and ongoing modification to increase usability.^104^ It has also demonstrated responsiveness to reversal of bowel damage following anti-TNFα therapy.^105^ Although less accurate than MRE for exploring some parts of the intestinal tract, especially the proximal small bowel, intestinal US has significant advantages in terms of greater availability, lower costs, minor invasiveness, and greater patient acceptability.^41^ Two simple intestinal US disease activity indices, the simple IUS score and the IBUS-SAS score, have been developed and validated.^106–108^

### 5.4. Cross-sectional measures using intestinal US as endpoints

There have been few studies measuring the effect of treatments on disease activity using intestinal US for cross-sectional imaging in UC. Improvements in BWT and vascularization have been observed following treatment in 3 prospective studies of various sizes.^80–82^

### 5.5. Composite endpoints of deep remission and disease clearance

While deep remission is broadly described as concurrent clinical and endoscopic remission or mucosal healing, there is no definitive, precise definition. Rates of deep remission (defined using various combinations of endoscopic and clinical outcomes) ranged from 27.0% to 58.5% after 1 year of vedolizumab or anti-tumor necrosis factorα (anti-TNFα) treatment.^83–85^ In a weighted, propensity score analysis, vedolizumab-treated patients were more likely to achieve deep remission and steroid-free deep remission than those receiving anti-TNFα treatments.^86^ There have been few studies with biologics or small molecules reporting on the more stringent composite outcome of disease clearance, which includes concurrent clinical, endoscopic, and histological remission. Rates of disease clearance of between 16.3% and 29.2% have been reported.^87,88^ A post hoc analysis of the VARSITY study suggests that patients treated with vedolizumab were more successful at reaching this endpoint than those treated with adalimumab.^87^ Achieving disease clearance has been associated with a significantly lower risk of escalation of medical therapy, UC‐related hospitalization, UC‐related surgery, colorectal dysplasia/neoplasia, and death.^88^

## 6. Clinical studies employing novel endpoints in CD

Data from novel outcome measures used in clinical studies of patients with CD are shown in Table 4.

**Table 4.** Studies of biologic and small molecule therapies using novel IBD outcomes in CD

Study	Treatment	Key study details and findings
Histological remission/healing		
VERSIFY—Phase 3b, prospective, open-label, single-group study^109^	Vedolizumab	Histologic remission = no neutrophils in the epithelium in patients with neutrophils in the epithelium at baseline (exploratory endpoint)Primary study population (*n* = 101) patients with inadequate/loss of response/intolerance to standard CD treatments (CS, immunosuppressants or anti-TNFα agents) treated with vedolizumab for up to 26 weeksSubstudy population comprised 56 patients treated with vedolizumab for up to 52 weeksHistological remission with vedolizumab treatment Week 26 in 14/92 (15.2% [95% CI, 8.6-24.2]) patients in the primary study Week 52 in 11 of 55 patients (20% [95% CI, 10.4-33.0]) in the substudy
LOVE-CD—Prospective study at tertiary centers in Belgium and The Netherlands^110^	Vedolizumab	Histological remission = GS < 3.1 (absence of neutrophils in the epithelium) or RHI ≤ 6 (absence of granulocyte in mucosal biopsies)Study population included both anti-TNFα-experienced and -naïve patients with CD and ulcerations at baseline endoscopy, analysis of paired biopsies from all segments at baseline and week 26 where active inflammation (GS ≥ 3 or RHI > 7) was present at baselineHistological remission with vedolizumab treatment Week 26 in 64% (43/67) of patients based on GS and 66% (37/56) of patients based on RHI scores
EXTEND—Multicenter, randomized, double-blind, placebo-controlled clinicaltrial^111^	Adalimumab	Histological healing = GHAS ≤ 2 assessed in the ileum and colonAmong patients with CD receiving maintenance adalimumab treatment and who had a colon/ileum GHAS score ≥ 3 at baseline,Histological remission with adalimumab Week 52 histological remission in the colon (*n* = 53) achieved by 28.3% of patients and in the ileum (*n* = 33) by 21.2%
Post hoc analysis of Phase 2 SERENITY trial^112^	Mirikizumab	Histologic remission = absence of mucosal neutrophils or epithelial damage3 histology scoring systems employed: RHI, modified GHAS, and active GHASAt week 12, rates of histological remission in all intestinal segments were greater in mirikizumab-treated patients 26% (13/50) than placebo-treated patients 6% (3/49); *P* < .01At week 52, 13%-31% of mirikizumab-treated patients achieved histological remission in all intestinal segments
Transmural remission/healing		
VERSIFY—Phase 3b, open-label, single-group study using MRE (MaRIA)—exploratory endpoint^109^	Vedolizumab	Radiologic remission = MaRIA scores < 7 in all segments, or MaRIA scores < 11 in all bowel segments in those patients with scores of ≥7 or ≥11 in at least 1 segment at baseline, respectively (exploratory endpoint)MRE evaluations performed in 32 patients (primary study) treated with vedolizumab for up to 26 weeks and 21 patients (substudy) treated with vedolizumab for up to 52 weeksMaRIA-7 radiologic remissionWeek 26 in 7/32 patients (21.9%; 95% CI, 9.3-40.0)Week 52 in 8/21 patients (38.1%; 95% CI, 18.1-61.6)MaRIA-11 radiologic remissionWeek 26 in 11/32 (34.4%; 95% CI, 18.6-53.2)Week 52 in 9/21 (42.9%; 95% CI, 21.8-66.0)
Real-world prospective observational study using MRI or intestinal US^113^	Ustekinumab	Transmural healing = complete healing of all layers of the bowel as assessed by MRI or normal US examination with a decrease in BWT to values ≤ 3 mmStudy included 92 ustekinumab-treated patients. Transmural healing at week 52 assessed by MRI in 40 patients and intestinal US in 35 patients as a secondary endpoint.Transmural healing in ustekinumab-treated patients Week 52 transmural healing (MRI) in 15/40 (37.5%) patients Week 52 transmural healing (intestinal US) in 11/35 (31.4%) patients
Retrospective observational single-center study using MRE (MaRIA)^114^	Ustekinumab	Transmural healing on MRE = BWT ≤ 3 mm without any signs of inflammation (ie, ulceration, edema, diffusion-weighted hyperintensity, and increased contrast enhancement)Study included 37 ustekinumab-treated patients. The primary outcome was transmural healing at week 26 evaluated using MRE, baseline predictors of transmural healing at week 26 analyzed as a secondary outcomeTransmural healing in ustekinumab-treated patients Week 26, transmural healing in 7/37 patients (18.9%) Week 26, decreased baseline BWT (OR 0.29 [95% CI, 0.1-0.9]; *P* = .035) and increased apparent diffusion coefficient (OR 3.0 [95% CI, 1.0-8.9]; *P* = .048) were independent predictors for transmural healing (multivariate analysis)
Real-world prospective single-center study using MRE (sMaRIA)^115^	Infliximab, adalimumab, ustekinumab, and vedolizumab	Transmural remission = sMaRIA score of the most affected segment < 2 on MREStudy included 134 patients treated with biologic agents (induction and 1 year of maintenance); outcomes were compared between patients achieving or not achieving transmural remissionTransmural remission rate at 1 year was 40% (54/134 patients)After a median of 30 months, 43 (32%) patients were hospitalized Patients with transmural remission had a lower risk of hospitalization than those without remission (*P* < .01) Hospitalization-free rates were 96%, 94%, and 91% after 12, 24, and 36 months follow-up, respectively. Adjusted HR of transmural remission for predicting hospitalization was 0.11 (95% CI, 0.04-0.32); *P* < .01 Patients with transmural remission had a lower risk of surgery than those without (*P* < .01) Adjusted HR of transmural remission for predicting surgery was 0.02 (95% CI, 0.00-0.92); *P* = .04
Observational multicenter study in France using MRE (MaRIA)^116^	Anti-TNFα	Transmural response = ≥25% improvement in MaRIA scoreAnalysis of 46 infliximab-treated patients with MRI data at baseline, and weeks 12 and 52 following anti-TNFα treatmentTransmural response at week 12 (OR 4.2 95% CI, 1.3-13.3; *P* = .015) was predictive of corticosteroid-free remission at week 52
Real-world prospective observational single center study using intestinal US^117^	Anti-TNFα	Transmural healing = BWT ≤ 3 mm measured using intestinal US1-year clinical outcomes evaluated in 218 patients treated with anti-TNFα therapies (infliximab or adalimumab) for 2 years compared in patients achieving vs not achieving transmural healingTransmural healing in 68/218 patients (31.2%) after 2 years of anti‐TNFα treatment At 1-year follow-up, transmural healing associated with a higher rate of steroid-free clinical remission than mucosal healing alone and no healing, 95.58%, 75%, and 41.11%, respectively (*P* < .001) Hospitalization during 1-year follow‐up was significantly lower in the transmural healing group (8.8%) than the mucosal healing group (28.3%) and no healing group (66.6%; *P* < .001) and occurred later than in patients with mucosal healing (HR 0.88 [95% CI, 0.69-0.95]; *P* = .007) or no healing (HR 0.58 [95% CI, 0.44-0.75]; *P* = .008) Need for surgery during 1-year follow-up was significantly lower in patients with transmural healing vs and those with mucosal healing or no healing (*P* = .007) and occurred later than patients with mucosal healing (HR 0.94 [95% CI, 0.84-0.98]; *P* = .009) or no healing (HR 0.79 [95% CI, 0.62-0.84]; *P* = .006) At 1-year follow-up, transmural healing was an independent risk factor for steroid-free clinical remission, and reduced need for hospitalization and surgery
Real-world prospective multicenter study using intestinal US^118^	Adalimumab, infliximab, vedolizumab, and ustekinumab	Transmural healing = normalization of intestinal US parametersAnalysis included 188 patients with CD treated with a biologic (adalimumab *n* = 103, infliximab *n* = 31, vedolizumab *n* = 24, and ustekinumab *n* = 30) and followed up for 1 year, intestinal US was performed at baseline and months 3, 6, and 12`Transmural healing rate at Months 3, 6, and 12 was 16.4%, 24.5%, and 27.5% Transmural healing at 12 months: 37% infliximab-treated patients, 27.2% vedolizumab-treated, 26.5% adalimumab-treated, and 20% ustekinumab-treated
MORE—Prospective multicenter study in China, using intestinal US^119^	Infliximab	Transmural healing = BWT ≤ 3.0 mm, preserved BWS, DCS 0-1, and the absence of i-fat in the most affected segment identified by intestinal USStudy included 129 patients who received infliximab for ≥44-52 weeks. Intestinal US performed at baseline, weeks 14-26, and post-maintenance weeks 44-56.Weeks 44-56 49/129 (38.0%) of infliximab-treated patients achieved transmural healing Multivariate analysis of baseline intestinal US factors identified the presence of i-fat at baseline as the best independent negative predictor for transmural healing (adjusted OR 0.57 95% CI, 0.38-0.87; *P* = .008) High BWT after induction was the best independent post-induction negative predictor for transmural healing (OR 0.24 95% CI, 0.14-0.42; *P* < .001)
STARDUST—multicenter, phase 3b randomized study, with intestinal US substudy^120^	Ustekinumab	Transmural remission = ≥25% BWT reduction from baseline and normalization of all intestinal US parametersSubstudy evaluated intestinal US parameters for 77 ustekinumab-treated patients with intestinal US assessments for exploratory analysisWeek 48 Transmural remission achieved in 24.1% of ustekinumab-treated patients (*n* = 54)
Deep remission		
Exploratory analysis of data from randomized, double-blind controlled study EXTEND^73^	Adalimumab	Deep remission = absence of mucosal ulceration plus clinical remission (CDAI < 150)Rates of deep remission (secondary study outcome) compared between the continuous adalimumab and adalimumab induction/placebo treatment groups at weeks 12 and 52Deep remission rates for continuous adalimumab vs adalimumab induction/placebo Week 12: 10/62 (16%) vs 6/61 (10%) (*P* = .34) Week 52: 12/62 (19%) vs 0/61 (0%) (*P* < .001) Patients with early deep remission by week 12 (*n* = 11) vs without early deep remission (*n* = 53) Fewer hospitalizations 0/11(0%) vs 9/53 (17%) Fewer CD-related surgeries 0/11 (0%) vs 5/53 (9%)
Real-world multicenter retrospective cohort study conducted in Scotland^121^	Ustekinumab	Deep remission = complete resolution of CD-related symptoms on PGA in the absence of CS + absence of mucosal ulceration/erosions on ileocolonoscopyAnalysis of deep remission (secondary endpoint) included 123 ustekinumab-treated patientsCumulative rates of deep remission 6 months 8.5% (*n* = 68) 12 months: 19.3% (*n* = 19)
Propensity matched retrospective analysis of data from 2 referral centers in France^122^	Ustekinumab and vedolizumab	Deep remission = CS-free clinical remission + deep biological remission of fecal calprotectin < 100 μg/g) at week 14 and week 24Analysis of deep remission (secondary endpoint) included 87 ustekinumab-treated and 45 vedolizumab-treated patients. Propensity score matching and inverse probability weighting (IPTW) were applied to minimize baseline group differencesDeep remission after IPTW ustekinumab vs vedolizumab Week 14: 17.9% vs 5.7% (*P* = .047) Week 24: 26.6% vs 16.1% (*P* = .58)
Patient-reported outcomes		
Post hoc analysis Phase 2b trials^123^	Tofacitanib	PRO2-75 clinical remission = the sum of SF score and AP score < 75PRO3-80 clinical remission = the sum of SF score, AP score, and general well-being score < 80Post hoc analyses of PRO endpoints at week 8 with non-responder imputation included 180 patients treated with tofacitinib 5 mg (*n* = 85), 10 mg (*n* = 86), or placebo (*n* = 90)Clinical remission at week 8 for tofacitinib 5 mg, 10 mg and placebo treatment groups PRO2-75: 50/85 (58.8%), 48/86 (55.8%), and 36/90 (40.0%) (*P* < .05 for tofacitinib 5 mg and 10 mg vs placebo) PRO3-80: 33/85 (38.8%), 31/86 (36.1%), and 22/90 (24.4%) (*P* < .05 for tofacitinib 5 mg vs placebo)
CELEST Phase 2 dose-ranging study^124^	Upadac itinib	PRO2 clinical remission = average daily SF of ≤1.5 and AP score of ≤1.0, with neither worse than the baseline value, was a week 16 PRO2 clinical remission was evaluated as co-primary endpoint with endoscopic remission. Study included 220 patients randomized to receive placebo (*n* = 37), or upadacitinib 3 mg BID (*n* = 39), 6 mg BID (*n* = 37), 12 mg BID (*n* = 36), or 24 mg BID (*n* = 36) or 24 mg QD (*n* = 35)Week 16 PRO2 clinical remission rates were 13%, 27%, 11%, 22%, and 14% for upadacitinib 3 mg BID, 6 mg BID and 12 mg BID, 24 mg BID, 24 mg QD, 11% for placebo.

Abbreviations: aHR, adjusted hazard ratio; AP, abdominal pain; BID, twice daily; BWS, bowel wall stratification; BWT, bowel wall thickness; CD, Crohn’s disease; CDAI, Crohn’s disease activity index; DCS, color Doppler signal; CI, confidence interval; CS, corticosteroid; GHAS, global histologic disease activity score; GS, Goebes Score; IBD, inflammatory bowel disease; i-fat, inflammatory mesenteric fat; QD, once daily; MaRIA, Magnetic Resonance Index of Activity for CD; MRE, magnetic resonance enterography; MRI, magnetic resonance imaging; PGA, Physicians Global Assessment; PRO, patient-reported outcome; RHI, Robarts Histology Index; SF, stool frequency; sMaRIA, simplified Magnetic Resonance Index of Activity for CD; TNFα, tumor necrosis factor alpha; US, ultrasound.

### 6.1. Novel PROs as endpoints

The novel interim PROs for assessing clinical remission in patients with CD, PRO2, and PRO3 are now used as endpoints in Phase 2 clinical trials of small molecules such as tofacitinib and upadacitinib. In the Phase 2 tofacitinib study, PRO2-75 and PRO3-80 were post hoc exploratory endpoints, while PRO2 was the co-primary endpoint in CELEST, the dose-ranging study of upadacitinib.^123,124^ Tofacitinib 5 mg was significantly more effective than placebo at inducing clinical remission as measured by the PRO2-75 and PRO3-80 outcome measures.^123^ Higher rates of clinical remission at week 16 were achieved with some but not all doses of upadacitinib vs placebo in the CELEST study.^124^

### 6.2. Histological remission/healing as a novel endpoint

Histological remission in patients with CD has been measured under different trial designs and using a range of indices and definitions in various patient populations treated with biological agents. Reported rates of histological remission ranged from 15% to 66% at week 24 and from 13% to 31% at week 52.^109–112^

### 6.3. Transmural healing as a novel endpoint

A systematic review published by Geyl and colleagues in 2021 reported rates of transmural healing in CD after anti-TNFα treatment (assessed using MRE, bowel US, and CTE, 897 patients in 10 studies) ranging from 14% to 42%.^70^Table 4 shows data on transmural healing with other biologic agents and data on anti-TNFα agents published after 2021 that were not included in the Geyl review; rates of transmural healing assessed using MRE, under various definitions of healing, ranged from 18.9% to 40% after 6-12 months of treatment.^109,113–115^ For intestinal US, transmural healing rates during maintenance therapy (week 44 up to 2 years of treatment) ranged from 24% to 38%.^117–120^ Transmural healing was associated with a higher rate of CS-free remission and a lower risk of hospitalization and surgery, and was an independent risk factor for CS-free clinical remission at 1 year.^115–117^

On MRE, parameters of decreased baseline BWT and increased apparent diffusion coefficient were independently associated with a higher likelihood of transmural healing.^115^ With intestinal US assessment, parameters such as the presence of inflammatory mesenteric fat at baseline and greater BWT post-induction were found to be negative predictors of transmural healing.^119^

### 6.4. Deep remission as a composite endpoint

Similar 1-year deep remission rates of 19% (where deep remission was defined as clinical remission plus mucosal healing) were achieved in patients treated with adalimumab in the EXTEND clinical trial or ustekinumab in a real-world retrospective study.^73,121^ A propensity score-matched analysis comparing rates of deep remission (defined as clinical and deep biologic remission) found no difference in 6-month rates of deep remission between ustekinumab and vedolizumab treatments.^122^

## 7. Future considerations

Future aspirational treatment targets for patients with IBD include intestinal barrier healing and molecular healing. Confocal laser endomicroscopy (CLE) is a high-resolution imaging technology that enables functional assessment of the integrity of the intestinal barrier. Pilot studies using CLE have indicated that barrier dysfunction in patients with IBD correlates with ongoing bowel symptoms and disease relapse.^125–127^ A large prospective study (ERIca) has recently shown that barrier healing is a better prognostic indicator than either endoscopic or histologic remission, alone or in combination, for forecasting the occurrence of major clinical events in both UC and CD. The study, by Rath and colleagues, lends support for considering the analysis of intestinal barrier function as a future treatment target in clinical trials.^128^

Molecular healing is the concept of restoring the specific inflammatory pathways involved in the etiopathogenesis of IBD. A biopsy molecular inflammation score (bMIS) and a circulating biomarker (cirMIS) gut inflammation score have been developed for the assessment of inflammatory markers. The bMIS may enable a more objective, granular, and sensitive measure of disease activity in IBD patients while the cirMIS provides a less invasive blood test for assessing disease activity. In an evaluation of these tools, both bMIS and cirMIS were strongly associated with clinical, endoscopic, and histological disease activity indices and both were responsive to IBD treatment. In addition, in patients considered macroscopically and microscopically “normal,” but with residual high bMIS/cirMIS (in UC) or high cirMIS (in CD) levels, rates of relapse were greater. These data support the hypothesis that residual molecular inflammation may predict relapse. Targeting inflammation that may persist at the molecular level, even in the presence of endoscopically or histologically normal mucosa, could present a future treatment target in IBD.

## 8. Conclusions

Over the past 2 decades, targeted therapies, including biologic agents and small molecules, have dramatically changed the treatment landscape and improved quality of life for people with IBD.

The ultimate goal of IBD treatment is to modify the course of the disease, so as to prevent disease extension in UC or bowel damage in CD and subsequent disability. This involves selecting optimal treatments, close monitoring according to appropriate therapeutic targets, and therapeutic adjustments throughout the disease course. It is therefore imperative to have an evidence base for novel outcomes showing that they reliably reflect modifications to the pathophysiological course of IBD and are predictive for reduced risk of complications and morbidities.

Endpoints have evolved over the years to become more stringent. In UC, histological remission and the composite histological-endoscopic endpoint have been associated with clinical, symptomatic, and CS-free remission, but data on indicators of disease modification (such as risk of hospitalization, UC-related surgery, or neoplasia) are still required. Disease clearance in UC has been associated with a significantly lower risk of UC‐related hospitalization, UC‐related surgery, colorectal dysplasia/neoplasia, and death; however, a consensus definition of disease clearance is lacking. In CD, transmural healing is predictive of a reduced need for hospitalization and surgery but there is a need for clarification of definitions using MRE or intestinal US. One further challenge is that current IBD management and treatments routinely achieve rates of only 40% for histological healing in UC and transmural healing in CD. Newer treatments and management strategies such as treating earlier in the disease course may allow these targets to be achieved by a larger proportion of patients. For these new, deeper endpoints, evidence for their superiority is heavily reliant on observational data and retrospective analysis. Evidence from disease modification trials is needed which aim to confirm the impact of current and novel outcomes on the course of IBD, including those recommended in the SPIRIT guidelines (Table 1). Data readouts from studies like VERDICT^47^ (NCT04259138) and VECTORS (NCT06257706) may be useful.

## Data Availability

The data underlying this article are available in the article.

## References

[1] Ng SC , Shi HY, Hamidi N, et al Worldwide incidence and prevalence of inflammatory bowel disease in the 21st century: a systematic review of population-based studies. Lancet. 2017;390:2769–2778. 29050646 10.1016/S0140-6736(17)32448-0

[2] Kaplan GG. The global burden of IBD: from 2015 to 2025. Nat Rev Gastroenterol Hepatol. 2015;12:720–727. 26323879 10.1038/nrgastro.2015.150

[3] Torres J , Mehandru S, Colombel JF, Peyrin-Biroulet L. Crohn’s disease. Lancet. 2017;389:1741–1755. 27914655 10.1016/S0140-6736(16)31711-1

[4] Ungaro R , Mehandru S, Allen PB, Peyrin-Biroulet L, Colombel JF. Ulcerative colitis. Lancet. 2017;389:1756–1770. 27914657 10.1016/S0140-6736(16)32126-2 PMC6487890

[5] IsHak WW , Pan D, Steiner AJ, et al Patient-reported outcomes of quality of life, functioning, and GI/psychiatric symptom severity in patients with inflammatory bowel disease (IBD). Inflamm Bowel Dis. 2017;23:798–803. 28301432 10.1097/MIB.0000000000001060

[6] Ding Z , Muser E, Izanec J, Lukanova R, Kershaw J, Roughley A. Work-related productivity loss and associated indirect costs in patients with Crohn’s disease or ulcerative colitis in the United States. Crohns Colitis 360. 2022;4:otac023. 36777416 10.1093/crocol/otac023 PMC9802455

[7] Le Berre C , Ananthakrishnan AN, Danese S, Singh S, Peyrin-Biroulet L. Ulcerative colitis and Crohn’s disease have similar burden and goals for treatment. Clin Gastroenterol Hepatol. 2020;18:14–23. 31301452 10.1016/j.cgh.2019.07.005

[8] Turner D , Ricciuto A, Lewis A, et al; International Organization for the Study of IBD. STRIDE-II: an update on the Selecting Therapeutic Targets in Inflammatory Bowel Disease (STRIDE) initiative of the International Organization for the Study of IBD (IOIBD): determining therapeutic goals for treat-to-target strategies in IBD. Gastroenterology. 2021;160:1570–1583. 33359090 10.1053/j.gastro.2020.12.031

[9] Le Berre C , Peyrin-Biroulet L; SPIRIT-IOIBD Study Group. Selecting end points for disease-modification trials in inflammatory bowel disease: the SPIRIT consensus from the IOIBD. Gastroenterology. 2021;160:1452–1460.e21. 33421515 10.1053/j.gastro.2020.10.065

[10] Food and Drug Administration. Crohn’s disease: developing drugs for treatment guidance for industry. 2022. Accessed July 11, 2024. https://www.fda.gov/media/158001/download

[11] Food and Drug Administration. Ulcerative colitis: developing drugs for treatment guidance for industry. 2022. Accessed July 11, 2024. https://www.fda.gov/media/158016/download

[12] European Medicines Agency. Guideline on the development of new medicinal products for the treatment of Crohn’s disease. 2018. Accessed July 11, 2024. https://www.ema.europa.eu/en/documents/scientific-guideline/guideline-development-new-medicinal-products-treatment-crohns-disease-revision-2_en.pdf

[13] European Medicines Agency. Guideline on the development of new medicinal products for the treatment of ulcerative colitis. 2018. Accessed July 11, 2024. https://www.ema.europa.eu/en/documents/scientific-guideline/guideline-development-new-medicinal-products-treatment-ulcerative-colitis-revision-1_en.pdf

[14] Khanna R , Bressler B, Levesque BG, et al; REACT Study Investigators. Early combined immunosuppression for the management of Crohn’s disease (REACT): a cluster randomised controlled trial. Lancet. 2015;386:1825–1834. 26342731 10.1016/S0140-6736(15)00068-9

[15] Jairath A , Khanna R, Guizzetti L, et al OP110. A cluster-randomised controlled trial of an enhanced treatment algorithm for the management of Crohn’s disease: REACT-2. United European Gastroenterol J. 2022;10:6–184.

[16] Ma C , Hanzel J, Panaccione R, et al; CORE-IBD Collaborators. CORE-IBD: a multidisciplinary international consensus initiative to develop a core outcome set for randomized controlled trials in inflammatory bowel disease. Gastroenterology. 2022;163:950–964. 35788348 10.1053/j.gastro.2022.06.068

[17] Jairath V , Khanna R, Zou GY, et al Development of interim patient-reported outcome measures for the assessment of ulcerative colitis disease activity in clinical trials. Aliment Pharmacol Ther. 2015;42:1200–1210. 26388424 10.1111/apt.13408

[18] Dulai PS , Jairath V, Khanna R, et al Development of the symptoms and impacts questionnaire for Crohn’s disease and ulcerative colitis. Aliment Pharmacol Ther. 2020;51:1047–1066. 32319120 10.1111/apt.15726 PMC7317756

[19] Higgins PDR , Harding G, Revicki DA, et al Development and validation of the ulcerative colitis patient-reported outcomes signs and symptoms (UC-pro/SS) diary. J Patient Rep Outcomes. 2018;2:26. 10.1186/s41687-018-0049-2 PMC5976680 29888745

[20] Dubinsky M , Bleakman AP, Panaccione R, et al Bowel urgency in ulcerative colitis: current perspectives and future directions. Am J Gastroenterol. 2023;118:1940–1953. 37436151 10.14309/ajg.0000000000002404 PMC10617668

[21] Sninsky J , Barnes E, Zhang X, Long M. P075 Urgency and its association with quality of life and clinical outcomes in ulcerative colitis patients. Am J Gastroenterol. 2021;116:S19–S20. 10.14309/ajg.0000000000001685 PMC9064909 35169109

[22] Schreiber S , Hunter Gibble T, Panaccione R, et al Patient and health care professional perceptions of the experience and impact of symptoms of moderate-to-severe Crohn’s disease in US and Europe: results from the cross-sectional CONFIDE study. Dig Dis Sci. 2024;69:2333–2344. 38700629 10.1007/s10620-024-08434-5 PMC11258049

[23] Dubinsky MC , Irving PM, Panaccione R, et al Incorporating patient experience into drug development for ulcerative colitis: development of the Urgency Numeric Rating Scale, a patient-reported outcome measure to assess bowel urgency in adults. J Patient Rep Outcomes. 2022;6:31. 35362902 10.1186/s41687-022-00439-w PMC8975984

[24] Lenfant M , Verstockt B, Sabino J, Vermeire S, Ferrante M. The assessment of segmental healing by the Modified Mayo Endoscopic Score (MMES) complements the prediction of long-term clinical outcomes in patients with ulcerative colitis. Aliment Pharmacol Ther. 2024;59:64–70. 37843544 10.1111/apt.17753

[25] Vespa E , D’Amico F, Sollai M, et al Histological scores in patients with inflammatory bowel diseases: the state of the art. J Clin Med. 2022;11:939. 35207211 10.3390/jcm11040939 PMC8880199

[26] Kevans D , Kirsch R, Dargavel C, Kabakchiev B, Riddell R, Silverberg MS. Histological markers of clinical relapse in endoscopically quiescent ulcerative colitis. Inflamm Bowel Dis. 2020;26:1722–1729. 31883337 10.1093/ibd/izz308 PMC8243631

[27] González-Partida I , Martínez-Lozano H, González-Lois C, et al Histological inflammatory activity can predict endoscopic relapse in patients with ulcerative colitis who have achieved mucosal healing. Eur J Gastroenterol Hepatol. 2021;33:e796–e802. 34334707 10.1097/MEG.0000000000002258

[28] Shehab M , Al Akram S, Hassan A, Alrashed F, Jairath V, Bessissow T. Histological disease activity as predictor of clinical relapse, hospitalization, and surgery in inflammatory bowel disease: systematic review and meta-analysis. Inflamm Bowel Dis. 2023;30:563–572. 10.1093/ibd/izad119 37541185

[29] Park S , Abdi T, Gentry M, Laine L. Histological disease activity as a predictor of clinical relapse among patients with ulcerative colitis: systematic review and meta-analysis. Am J Gastroenterol. 2016;111:1692–1701. 27725645 10.1038/ajg.2016.418

[30] Bryant RV , Burger DC, Delo J, et al Beyond endoscopic mucosal healing in UC: histological remission better predicts corticosteroid use and hospitalisation over 6 years of follow-up. Gut. 2016;65:408–414. 25986946 10.1136/gutjnl-2015-309598

[31] Battat R , Duijvestein M, Guizzetti L, et al Histologic healing rates of medical therapies for ulcerative colitis: a systematic review and meta-analysis of randomized controlled trials. Am J Gastroenterol. 2019;114:733–745. 30694863 10.14309/ajg.0000000000000111

[32] Wetwittayakhlang P , Lontai L, Gonczi L, et al Treatment targets in ulcerative colitis: is it time for all in, including histology? J Clin Med. 2021;10:5551. 34884252 10.3390/jcm10235551 PMC8658443

[33] Pai RK , Jairath V, Vande Casteele N, Rieder F, Parker CE, Lauwers GY. The emerging role of histologic disease activity assessment in ulcerative colitis. Gastrointest Endosc. 2018;88:887–898. 30142351 10.1016/j.gie.2018.08.018

[34] Jairath V , Peyrin-Biroulet L, Zou G, et al Responsiveness of histological disease activity indices in ulcerative colitis: a post hoc analysis using data from the TOUCHSTONE randomised controlled trial. Gut. 2019;68:1162–1168. 30076171 10.1136/gutjnl-2018-316702

[35] Ma C , Sedano R, Almradi A, et al An international consensus to standardize integration of histopathology in ulcerative colitis clinical trials. Gastroenterology. 2021;160:2291–2302. 33610533 10.1053/j.gastro.2021.02.035 PMC8851891

[36] Rath T , Atreya R, Neurath MF. Is histological healing a feasible endpoint in ulcerative colitis? Expert Rev Gastroenterol Hepatol. 2021;15:665–674. 33481635 10.1080/17474124.2021.1880892

[37] Allocca M , Fiorino G, Bonovas S, et al Accuracy of Humanitas ultrasound criteria in assessing disease activity and severity in ulcerative colitis: a prospective study. J Crohns Colitis. 2018;12:1385–1391. 30085066 10.1093/ecco-jcc/jjy107 PMC6260119

[38] Bots S , Nylund K, Löwenberg M, Gecse K, D’Haens G. Intestinal ultrasound to assess disease activity in ulcerative colitis: development of a novel UC-ultrasound index. J Crohns Colitis. 2021;15:1264–1271. 33411887 10.1093/ecco-jcc/jjab002 PMC8328285

[39] Allocca M , Filippi E, Costantino A, et al Milan ultrasound criteria are accurate in assessing disease activity in ulcerative colitis: external validation. United European Gastroenterol J. 2021;9:438–442. 10.1177/2050640620980203 PMC8259285 33349199

[40] Rimola J , Torres J, Kumar S, Taylor SA, Kucharzik T. Recent advances in clinical practice: advances in cross-sectional imaging in inflammatory bowel disease. Gut. 2022;71:2587–2597. 35927032 10.1136/gutjnl-2021-326562 PMC9664122

[41] Mignini I , Maresca R, Ainora ME, et al Predicting treatment response in inflammatory bowel diseases: cross-sectional imaging markers. J Clin Med. 2023;12:5933. 37762874 10.3390/jcm12185933 PMC10532020

[42] Parkes G , Ungaro RC, Danese S, et al Correlation of mucosal healing endpoints with long-term clinical and patient-reported outcomes in ulcerative colitis. J Gastroenterol. 2023;58:990–1002. 37490069 10.1007/s00535-023-02013-7 PMC10522527

[43] Danese S , Roda G, Peyrin-Biroulet L. Evolving therapeutic goals in ulcerative colitis: towards disease clearance. Nat Rev Gastroenterol Hepatol. 2020;17:1–2. 31520081 10.1038/s41575-019-0211-1

[44] Zallot C , Peyrin-Biroulet L. Deep remission in inflammatory bowel disease: looking beyond symptoms. Curr Gastroenterol Rep. 2013;15:315. 23354742 10.1007/s11894-013-0315-7

[45] D’Amico F , Magro F, Siegmund B, et al; the end point cluster of the International Organization for the Study of Inflammatory Bowel Diseases (IOIBD). Disease clearance as a new outcome in ulcerative colitis: a systematic review and expert consensus. Inflamm Bowel Dis. 2023;30:1009–1017. 10.1093/ibd/izad159 37549104

[46] D’Amico F , Peyrin-Biroulet L, Danese S. Disease clearance in ulcerative colitis: is the ultimate therapeutic target? United European Gastroenterol J. 2023;11:717–719. 10.1002/ueg2.12436 PMC10576599 37401029

[47] Jairath V , Zou G, Wang Z, et al Determining the optimal treatment target in patients with ulcerative colitis: rationale, design, protocol and interim analysis for the randomised controlled VERDICT trial. BMJ Open Gastroenterol. 2024;11:e001218. 10.1136/bmjgast-2023-001218 PMC10870790 38336367

[48] Sandborn WJ , Vermeire S, Peyrin-Biroulet L, et al Etrasimod as induction and maintenance therapy for ulcerative colitis (ELEVATE): two randomised, double-blind, placebo-controlled, phase 3 studies. Lancet. 2023;401:1159–1171. 36871574 10.1016/S0140-6736(23)00061-2

[49] Khanna R , Zou G, D’Haens G, et al A retrospective analysis: the development of patient reported outcome measures for the assessment of Crohn’s disease activity. Aliment Pharmacol Ther. 2015;41:77–86. 25348809 10.1111/apt.13001

[50] Higgins PDR , Harding G, Leidy NK, et al Development and validation of the Crohn’s disease patient-reported outcomes signs and symptoms (CD-PRO/SS) diary. J Patient Rep Outcomes. 2018;2:24. 10.1186/s41687-018-0044-7 PMC5942337 29770803

[51] Pai RK , Jairath V. What is the role of histopathology in the evaluation of disease activity in Crohn’s disease? Best Pract Res Clin Gastroenterol. 2019;38–39:101601. 10.1016/j.bpg.2019.02.003 31327406

[52] Novak G , Parker CE, Pai RK, et al Histologic scoring indices for evaluation of disease activity in Crohn’s disease. Cochrane Database Syst Rev. 2017;7:CD012351. 28731502 10.1002/14651858.CD012351.pub2 PMC6483549

[53] Solitano V , Schaeffer DF, Hogan M, et al Reliability and responsiveness of histologic indices for the assessment of Crohn’s disease activity. Clin Gastroenterol Hepatol. 2024;22:1898–1907.e25. https://doi.org/10.1016/j.cgh.2023.11.032 38056798

[54] Roseira J , Santiago M, Estevinho MM, et al Impact of Crohn’s disease therapies on histology in randomized controlled trials: systematic review with meta-analysis. Inflamm Bowel Dis. 2023;29:1231–1243. 36250778 10.1093/ibd/izac203

[55] Mojtahed A , Khanna R, Sandborn WJ, et al Assessment of histologic disease activity in Crohn’s disease: a systematic review. Inflamm Bowel Dis. 2014;20:2092–2103. 25137418 10.1097/MIB.0000000000000155

[56] Shah SC , Colombel JF, Sands BE, Narula N. Systematic review with meta-analysis: mucosal healing is associated with improved long-term outcomes in Crohn’s disease. Aliment Pharmacol Ther. 2016;43:317–333. 26607562 10.1111/apt.13475

[57] Pray C , Narula NE. Editorial: Moving towards prognostic endoscopic scoring in IBD. Aliment Pharmacol Ther. 2024;59:132–133. 38085938 10.1111/apt.17787

[58] Narula N , Wong ECL, Colombel JF, et al Predicting endoscopic remission in Crohn’s disease by the modified multiplier SES-CD (MM-SES-CD). Gut. 2022;71:1078–1087. 33766910 10.1136/gutjnl-2020-323799

[59] Narula N , Wong ECL, Dulai PS, et al; CALM LTE investigators. Defining endoscopic remission in Crohn’s disease: MM-SES-CD and SES-CD thresholds associated with low risk of disease progression. Clin Gastroenterol Hepatol. 2024;22:1687–1696.e6. https://doi.org/10.1016/j.cgh.2024.02.009 38428709

[60] Le Berre C , Trang-Poisson C, Bourreille A. Small bowel capsule endoscopy and treat-to-target in Crohn’s disease: a systematic review. World J Gastroenterol. 2019;25:4534–4554. 31496630 10.3748/wjg.v25.i31.4534 PMC6710184

[61] Pennazio M , Rondonotti E, Despott EJ, et al Small-bowel capsule endoscopy and device-assisted enteroscopy for diagnosis and treatment of small-bowel disorders: European Society of Gastrointestinal Endoscopy (ESGE) Guideline—update 2022. Endoscopy. 2023;55:58–95. 36423618 10.1055/a-1973-3796

[62] Annese V , Daperno M, Rutter MD, et al; European Crohn's and Colitis Organisation. European evidence based consensus for endoscopy in inflammatory bowel disease. J Crohns Colitis. 2013;7:982–1018. 24184171 10.1016/j.crohns.2013.09.016

[63] Cotter J , Dias de Castro F, Magalhaes J, Moreira MJ, Rosa B. Validation of the Lewis score for the evaluation of small-bowel Crohn’s disease activity. Endoscopy. 2015;47:330–335. 25412092 10.1055/s-0034-1390894

[64] Gralnek IM , Defranchis R, Seidman E, Leighton JA, Legnani P, Lewis BS. Development of a capsule endoscopy scoring index for small bowel mucosal inflammatory change. Aliment Pharmacol Ther. 2008;27:146–154. 17956598 10.1111/j.1365-2036.2007.03556.x

[65] Gal E , Geller A, Fraser G, Levi Z, Niv Y. Assessment and validation of the new capsule endoscopy Crohn’s disease activity index (CECDAI). Dig Dis Sci. 2008;53:1933–1937. 18034304 10.1007/s10620-007-0084-y

[66] Niv Y , Gal E, Gabovitz V, Hershkovitz M, Lichtenstein L, Avni I. Capsule endoscopy Crohn’s disease activity index (CECDAIic or Niv Score) for the small bowel and colon. J Clin Gastroenterol. 2018;52:45–49. 27753700 10.1097/MCG.0000000000000720

[67] Niv Y , Ilani S, Levi Z, et al Validation of the capsule endoscopy Crohn’s disease activity index (CECDAI or Niv score): a multicenter prospective study. Endoscopy. 2012;44:21–26. 22125196 10.1055/s-0031-1291385

[68] Rosa B , Pinho R, de Ferro SM, Almeida N, Cotter J, Saraiva MM. Endoscopic scores for evaluation of Crohn’s disease activity at small bowel capsule endoscopy: general principles and current applications. GE Port J Gastroenterol. 2016;23:36–41. 28868428 10.1016/j.jpge.2015.08.004 PMC5580095

[69] Ben-Horin S , Lahat A, Ungar B, et al DOP29 Capsule endoscopy-guided proactive treatment versus standard treatment of patients with quiescent Crohn’s disease: the CURE-CD randomized controlled trial. J Crohns Colitis. 2024;18(Suppl 1):i125–i126.

[70] Geyl S , Guillo L, Laurent V, D’Amico F, Danese S, Peyrin-Biroulet L. Transmural healing as a therapeutic goal in Crohn’s disease: a systematic review. Lancet Gastroenterol Hepatol. 2021;6:659–667. 34090579 10.1016/S2468-1253(21)00096-0

[71] Rubin DT. Transmural healing in inflammatory bowel disease. Gastroenterol Hepatol (N Y). 2023;19:101–103. 36866113 PMC9972610

[72] Castiglione F , Imperatore N, Testa A, et al Exploring the concept of deep remission in Crohn’s disease: correlation between transmural healing and biomarkers. Therap Adv Gastroenterol. 2022;15. https://doi.org/10.1177/17562848221110643 PMC9310328 35898191

[73] Colombel JF , Rutgeerts PJ, Sandborn WJ, et al Adalimumab induces deep remission in patients with Crohn’s disease. Clin Gastroenterol Hepatol. 2014;12:414–422. 23856361 10.1016/j.cgh.2013.06.019

[74] Ungaro RC , Yzet C, Bossuyt P, et al Deep remission at 1 year prevents progression of early Crohn’s disease. Gastroenterology. 2020;159:139–147. 32224129 10.1053/j.gastro.2020.03.039 PMC7751802

[75] Raine T , Danese S, Schreiber S, et al DOP64 Steroid-sparing effect of risankizumab vs ustekinumab in patients with moderately to severely active Crohn’s disease: post hoc results from the phase 3b SEQUENCE trial. J Crohns Colitis. 2024;18:i190–i190. 10.1093/ecco-jcc/jjag104 PMC13387155 42479626

[76] Peyrin-Biroulet L , Loftus EV Jr., Colombel JF, et al Histologic outcomes with vedolizumab versus adalimumab in ulcerative colitis: results from an efficacy and safety study of vedolizumab intravenous compared to adalimumab subcutaneous in participants with ulcerative colitis (VARSITY). Gastroenterology. 2021;161:1156–1167.e3. 34144047 10.1053/j.gastro.2021.06.015

[77] Magro F , Pai RK, Kobayashi T, et al Resolving histological inflammation in ulcerative colitis with mirikizumab in the LUCENT induction and maintenance trial programmes. J Crohns Colitis. 2023;17:1457–1470. 37057827 10.1093/ecco-jcc/jjad050 PMC10588772

[78] Arijs I , De Hertogh G, Lemmens B, et al Effect of vedolizumab (anti-α4β7-integrin) therapy on histological healing and mucosal gene expression in patients with UC. Gut. 2018;67:43–52. 27802155 10.1136/gutjnl-2016-312293

[79] Li K , Marano C, Zhang H, et al Relationship between combined histologic and endoscopic endpoints and efficacy of ustekinumab treatment in patients with ulcerative colitis. Gastroenterology. 2020;159:2052–2064. 32853634 10.1053/j.gastro.2020.08.037

[80] Maaser C , Petersen F, Helwig U, et al; German IBD Study Group and the TRUST&UC Study Group. Intestinal ultrasound for monitoring therapeutic response in patients with ulcerative colitis: results from the TRUST&UC study. Gut. 2020;69:1629–1636. 31862811 10.1136/gutjnl-2019-319451 PMC7456734

[81] de Voogd F , van Wassenaer EA, Mookhoek A, et al Intestinal ultrasound is accurate to determine endoscopic response and remission in patients with moderate to severe ulcerative colitis: a longitudinal prospective cohort study. Gastroenterology. 2022;163:1569–1581. 36030056 10.1053/j.gastro.2022.08.038

[82] Goertz RS , Klett D, Wildner D, Atreya R, Neurath MF, Strobel D. Quantitative contrast-enhanced ultrasound for monitoring vedolizumab therapy in inflammatory bowel disease patients: a pilot study. Acta Radiol. 2018;59:1149–1156. 29345146 10.1177/0284185117752032

[83] Sandborn WJ , Colombel JF, Panaccione R, et al Deep remission with vedolizumab in patients with moderately to severely active ulcerative colitis: a GEMINI 1 post hoc analysis. J Crohns Colitis. 2019;13:172–181. 30285104 10.1093/ecco-jcc/jjy149 PMC6357899

[84] Muñoz-Villafranca C , Ortiz de Zarate J, Arreba P, et al Adalimumab treatment of anti-TNF-naïve patients with ulcerative colitis: deep remission and response factors. Dig Liver Dis. 2018;50:812–819. 29625907 10.1016/j.dld.2018.03.007

[85] Narula N , Peerani F, Meserve J, et al Vedolizumab for ulcerative colitis: treatment outcomes from the VICTORY Consortium. Am J Gastroenterol. 2018;113:1345. 29946178 10.1038/s41395-018-0162-0 PMC6445254

[86] Lukin D , Faleck D, Xu R, et al Comparative safety and effectiveness of vedolizumab to tumor necrosis factor antagonist therapy for ulcerative colitis. Clin Gastroenterol Hepatol. 2022;20:126–135. 33039584 10.1016/j.cgh.2020.10.003 PMC8026779

[87] Danese S , Schreiber S, Loftus E Jr., et al P271 Evolving targets in ulcerative colitis: defining disease clearance in the VARSITY study. J Crohns Colitis. 2021;15:S305–S305.

[88] D’Amico F , Fiorino G, Solitano V, et al Ulcerative colitis: Impact of early disease clearance on long-term outcomes—a multicenter cohort study. United European Gastroenterol J. 2022;10:775–782. 10.1002/ueg2.12288 PMC9486490 36107109

[89] Feagan BG , Lasch K, Lissoos T, et al Rapid response to vedolizumab therapy in biologic-naive patients with inflammatory bowel diseases. Clin Gastroenterol Hepatol. 2019;17:130–138.e7. 29857145 10.1016/j.cgh.2018.05.026

[90] Danese S , Sands BE, Abreu MT, et al Early symptomatic improvement after ustekinumab therapy in patients with ulcerative colitis: 16-week data from the UNIFI trial. Clin Gastroenterol Hepatol. 2022;20:2858–2867.e5. 35276329 10.1016/j.cgh.2022.02.050

[91] Danese S , Ferrante M, Feagan BG, et al Rapid and sustained symptom relief in patients with ulcerative colitis treated with filgotinib: data from the phase 2b/3 SELECTION trial. Am J Gastroenterol. 2023;118:138–147. 36113491 10.14309/ajg.0000000000001979 PMC9810009

[92] Hudesman DP , Torres J, Salese L, et al Long-term improvement in the patient-reported outcomes of rectal bleeding, stool frequency, and health-related quality of life with tofacitinib in the ulcerative colitis OCTAVE clinical program. Patient. 2023;16:95–103. 36336750 10.1007/s40271-022-00603-w PMC9911479

[93] Kappelman MD , Long MD, Zhang X, et al Comparing patient-reported outcomes among anti-TNF experienced patients with ulcerative colitis initiating vedolizumab versus tofacitinib. Crohns Colitis 360. 2023;5:otad031. 37350775 10.1093/crocol/otad031 PMC10284045

[94] Ghosh S , Sanchez Gonzalez Y, Zhou W, et al Upadacitinib treatment improves symptoms of bowel urgency and abdominal pain, and correlates with quality of life improvements in patients with moderate to severe ulcerative colitis. J Crohns Colitis. 2021;15:2022–2030. 34107013 10.1093/ecco-jcc/jjab099 PMC8684481

[95] Danese S , Tran J, D’Haens G, et al Upadacitinib induction and maintenance therapy improves abdominal pain, bowel urgency, and fatigue in patients with ulcerative colitis: a post hoc analysis of phase 3 data. Inflamm Bowel Dis. 2023;29:1723–1729. 36790041 10.1093/ibd/izad016 PMC10628919

[96] Dubinsky MC , Panaccione R, Lewis JD, et al Impact of bowel urgency on quality of life and clinical outcomes in patients with ulcerative colitis. Crohns Colitis 360. 2022;4:otac016. 36777426 10.1093/crocol/otac016 PMC9802402

[97] Peyrin-Biroulet L , Dubinsky MC, Sands BE, et al P407 Efficacy and safety of etrasimod in subjects with moderately to severely active isolated proctitis: a subgroup analysis of the phase 3 ELEVATE UC 52 and ELEVATE UC 12 trials. J Crohns Colitis. 2023;17:i536–i538.

[98] Li K , Friedman JR, Chan D, et al Effects of ustekinumab on histologic disease activity in patients with Crohn’s disease. Gastroenterology. 2019;157:1019–1031.e7. 31279870 10.1053/j.gastro.2019.06.037

[99] Jairath V , Zou G, Adsul S, et al DOP11 Disease clearance after 16 weeks of treatment with vedolizumab in patients with moderate to severe ulcerative colitis: an interim analysis from the VERDICT trial. J Crohns Colitis. 2024;18:i92–i93.

[100] Roseira J , Ventosa AR, de Sousa HT, Brito J. The new simplified MARIA score applies beyond clinical trials: a suitable clinical practice tool for Crohn’s disease that parallels a simple endoscopic index and fecal calprotectin. United European Gastroenterol J. 2020;8:1208–1216. 10.1177/2050640620943089 PMC7724527 32664824

[101] Ordás I , Rimola J, Alfaro I, et al Development and validation of a simplified magnetic resonance index of activity for Crohn’s disease. Gastroenterology. 2019;157:432–439.e1. 30953614 10.1053/j.gastro.2019.03.051

[102] Jairath V , Ordas I, Zou G, et al Reliability of measuring ileo-colonic disease activity in Crohn’s disease by magnetic resonance enterography. Inflamm Bowel Dis. 2018;24:440–449. 29361096 10.1093/ibd/izx040

[103] Hanžel J , Jairath V, Ma C, et al Responsiveness of magnetic resonance enterography indices for evaluation of luminal disease activity in Crohn’s disease. Clin Gastroenterol Hepatol. 2022;20:2598–2606. 35149220 10.1016/j.cgh.2022.01.055

[104] Pariente B , Torres J, Burisch J, et al Validation and update of the Lémann Index to measure cumulative structural bowel damage in Crohn’s disease. Gastroenterology. 2021;161:853–864.e13. 34052277 10.1053/j.gastro.2021.05.049 PMC8609534

[105] Fiorino G , Bonifacio C, Allocca M, et al Bowel damage as assessed by the Lémann index is reversible on anti-TNF therapy for Crohn’s disease. J Crohns Colitis. 2015;9:633–639. 25958059 10.1093/ecco-jcc/jjv080

[106] Novak KL , Kaplan GG, Panaccione R, et al A simple ultrasound score for the accurate detection of inflammatory activity in Crohn’s disease. Inflamm Bowel Dis. 2017;23:2001–2010. 28644185 10.1097/MIB.0000000000001174

[107] Novak KL , Nylund K, Maaser C, et al Expert consensus on optimal acquisition and development of the international bowel ultrasound segmental activity score [IBUS-SAS]: a reliability and inter-rater variability study on intestinal ultrasonography in Crohn’s disease. J Crohns Colitis. 2021;15:609–616. 33098642 10.1093/ecco-jcc/jjaa216 PMC8023841

[108] Sævik F , Eriksen R, Eide GE, Gilja OH, Nylund K. Development and validation of a simple ultrasound activity score for Crohn’s disease. J Crohns Colitis. 2021;15:115–124. 32504533 10.1093/ecco-jcc/jjaa112 PMC7805799

[109] Danese S , Sandborn WJ, Colombel JF, et al Endoscopic, radiologic, and histologic healing with vedolizumab in patients with active Crohn’s disease. Gastroenterology. 2019;157:1007–1018.e7. 31279871 10.1053/j.gastro.2019.06.038

[110] Löwenberg M , Vermeire S, Mostafavi N, et al Vedolizumab induces endoscopic and histologic remission in patients with Crohn’s disease. Gastroenterology. 2019;157:997–1006.e6. 31175865 10.1053/j.gastro.2019.05.067

[111] Reinisch W , Colombel JF, D’Haens G, et al Characterisation of mucosal healing with adalimumab treatment in patients with moderately to severely active Crohn’s disease: results from the EXTEND trial. J Crohns Colitis. 2017;11:425–434. 27815351 10.1093/ecco-jcc/jjw178 PMC5881717

[112] Magro F , Protic M, De Hertogh G, et al Effects of mirikizumab on histologic resolution of Crohn’s disease in a randomized controlled phase 2 trial. Clin Gastroenterol Hepatol. 2023;22:1878–1888.e10. https://doi.org/10.1016/j.cgh.2023.11.010 37993033

[113] Miranda A , Gravina AG, Cuomo A, et al Efficacy of ustekinumab in the treatment of patients with Crohn’s disease with failure to previous conventional or biologic therapy: a prospective observational real-life study. J Physiol Pharmacol. 2021;72:537–543. 10.26402/jpp.2021.4.05 34987127

[114] Zhou L , Hu C, Zhang R, et al Early transmural healing and its predictors assessed by magnetic resonance enterography in patients with Crohn’s disease receiving ustekinumab. Therap Adv Gastroenterol. 2023;16:10.1177/17562848231170947. 10.1177/17562848231170947 PMC10164861 37168404

[115] Takenaka K , Kawamoto A, Kitazume Y, et al Transmural remission characterized by high biologic concentrations demonstrates better prognosis in Crohn’s disease. J Crohns Colitis. 2022;17:855–862. 10.1093/ecco-jcc/jjac185 36527678

[116] Messadeg L , Hordonneau C, Bouguen G, et al Early transmural response assessed using magnetic resonance imaging could predict sustained clinical remission and prevent bowel damage in patients with Crohn’s disease treated with anti-tumour necrosis factor therapy. J Crohns Colitis. 2020;14:1524–1534. 32533769 10.1093/ecco-jcc/jjaa098

[117] Castiglione F , Imperatore N, Testa A, et al One-year clinical outcomes with biologics in Crohn’s disease: transmural healing compared with mucosal or no healing. Aliment Pharmacol Ther. 2019;49:1026–1039. 30854708 10.1111/apt.15190

[118] Calabrese E , Rispo A, Zorzi F, et al Ultrasonography tight control and monitoring in Crohn’s disease during different biological therapies: a multicenter study. Clin Gastroenterol Hepatol. 2022;20:e711–e722. 33775896 10.1016/j.cgh.2021.03.030

[119] Huang Z , Cheng W, Chao K, et al Baseline and postinduction intestinal ultrasound findings predict long-term transmural and mucosal healing in patients with Crohn’s disease. Inflamm Bowel Dis. 2024;30:1767–1775. https://doi.org/10.1093/ibd/izad251 37889843

[120] Kucharzik T , Wilkens R, D’Agostino MA, et al; STARDUST Intestinal Ultrasound Study Group. Early ultrasound response and progressive transmural remission after treatment with ustekinumab in Crohn’s disease. Clin Gastroenterol Hepatol. 2023;21:153–163.e12. 35842121 10.1016/j.cgh.2022.05.055

[121] Plevris N , Fulforth J, Siakavellas S, et al Real-world effectiveness and safety of ustekinumab for the treatment of Crohn’s disease: the Scottish ustekinumab cohort. J Gastroenterol Hepatol. 2021;36:2067–2075. 33381875 10.1111/jgh.15390

[122] Manlay L , Boschetti G, Pereira B, et al Comparison of short- and long-term effectiveness between ustekinumab and vedolizumab in patients with Crohn’s disease refractory to anti-tumour necrosis factor therapy. Aliment Pharmacol Ther. 2021;53:1289–1299. 33909920 10.1111/apt.16377

[123] Panés J , Sandborn WJ, Schreiber S, et al Tofacitinib for induction and maintenance therapy of Crohn’s disease: results of two phase IIb randomised placebo-controlled trials. Gut. 2017;66:1049–1059. 28209624 10.1136/gutjnl-2016-312735 PMC5532457

[124] Sandborn WJ , Feagan BG, Loftus EV Jr., et al Efficacy and safety of upadacitinib in a randomized trial of patients with Crohn’s disease. Gastroenterology. 2020;158:2123–2138.e8. 32044319 10.1053/j.gastro.2020.01.047

[125] Chang J , Leong RW, Wasinger VC, Ip M, Yang M, Phan TG. Impaired intestinal permeability contributes to ongoing bowel symptoms in patients with inflammatory bowel disease and mucosal healing. Gastroenterology. 2017;153:723–731.e1. 28601482 10.1053/j.gastro.2017.05.056

[126] Karstensen JG , Săftoiu A, Brynskov J, et al Confocal laser endomicroscopy: a novel method for prediction of relapse in Crohn’s disease. Endoscopy. 2016;48:364–372. 26583952 10.1055/s-0034-1393314

[127] Kiesslich R , Duckworth CA, Moussata D, et al Local barrier dysfunction identified by confocal laser endomicroscopy predicts relapse in inflammatory bowel disease. Gut. 2012;61:1146–1153. 22115910 10.1136/gutjnl-2011-300695 PMC3388727

[128] Rath T , Atreya R, Bodenschatz J, et al Intestinal barrier healing is superior to endoscopic and histologic remission for predicting major adverse outcomes in inflammatory bowel disease: the prospective ERIca Trial. Gastroenterology. 2023;164:241–255. 36279923 10.1053/j.gastro.2022.10.014

