# Correction to: Novel outcomes in inflammatory bowel disease

**DOI:** 10.1093/ecco-jcc/jjaf117

**Published:** 2025-10-01

This is a correction to: Vipul Jairath, Neeraj Narula, Ryan C Ungaro, Itzel Romo Bautista, Shashi Adsul, Novel outcomes in inflammatory bowel disease, *Journal of Crohn’s and Colitis*, Volume 19, Issue 4, April 2025, jjaf040, https://doi.org/10.1093/ecco-jcc/jjaf040

In the originally published version, in the graphical abstract, the text description for endoscopic outcome measures in UC incorrectly duplicated the text for symptomatic outcomes.

Additionally, in the text description for CD measures of endoscopic healing/remission, ‘disease activity on SBCE’ should have read ‘small bowel disease activity assessed by SBCE’.

The image has been updated online.

